# Dementia, dementia's risk factors and premorbid brain structure are concentrated in disadvantaged areas: National register and birth‐cohort geographic analyses

**DOI:** 10.1002/alz.13727

**Published:** 2024-03-14

**Authors:** Aaron Reuben, Leah S. Richmond‐Rakerd, Barry Milne, Devesh Shah, Amber Pearson, Sean Hogan, David Ireland, Ross Keenan, Annchen R. Knodt, Tracy Melzer, Richie Poulton, Sandhya Ramrakha, Ethan T. Whitman, Ahmad R. Hariri, Terrie E. Moffitt, Avshalom Caspi

**Affiliations:** ^1^ Department of Psychology and Neuroscience Duke University Durham North Carolina USA; ^2^ Department of Psychiatry and Behavioral Sciences Medical University of South Carolina Charleston South Carolina USA; ^3^ Department of Psychology University of Michigan Ann Arbor Michigan USA; ^4^ Centre for Methods and Policy Application in Society Sciences University of Auckland Auckland New Zealand; ^5^ Department of Geography, Environment, and Spatial Sciences Michigan State University East Lansing Michigan USA; ^6^ Department of Public Health University of Otago Wellington New Zealand; ^7^ Dunedin Multidisciplinary Health and Development Research Unit, Department of Psychology University of Otago Dunedin New Zealand; ^8^ Brain Health Research Centre, Department of Psychology University of Otago Dunedin New Zealand; ^9^ Department of Medicine University of Otago Christchurch New Zealand; ^10^ Department of Psychiatry and Behavioral Sciences Duke University Durham North Carolina USA; ^11^ King's College London, Social, Genetic, and Developmental Psychiatry Centre, Institute of Psychiatry, Psychology, & Neuroscience London UK; ^12^ PROMENTA, Department of Psychology University of Oslo Oslo Norway

**Keywords:** Alzheimer's disease and related dementias, Area Deprivation, Health disparities, Preventive medicine, Residential neighborhood socioeconomic status

## Abstract

**INTRODUCTION:**

Dementia risk may be elevated in socioeconomically disadvantaged neighborhoods. Reasons for this remain unclear, and this elevation has yet to be shown at a national population level.

**METHODS:**

We tested whether dementia was more prevalent in disadvantaged neighborhoods across the New Zealand population (*N* = 1.41 million analytic sample) over a 20‐year observation. We then tested whether premorbid dementia risk factors and MRI‐measured brain‐structure antecedents were more prevalent among midlife residents of disadvantaged neighborhoods in a population‐representative NZ‐birth‐cohort (*N* = 938 analytic sample).

**RESULTS:**

People residing in disadvantaged neighborhoods were at greater risk of dementia (HR per‐quintile‐disadvantage‐increase = 1.09, 95% confidence interval [CI]:1.08‐1.10) and, decades before clinical endpoints typically emerge, evidenced elevated dementia‐risk scores (CAIDE, LIBRA, Lancet, ANU‐ADRI, DunedinARB; β’s 0.31‐0.39) and displayed dementia‐associated brain structural deficits and cognitive difficulties/decline.

**DISCUSSION:**

Disadvantaged neighborhoods have more residents with dementia, and decades before dementia is diagnosed, residents have more dementia‐risk factors and brain‐structure antecedents. Whether or not neighborhoods causally influence risk, they may offer scalable opportunities for primary dementia prevention.

## 1 BACKGROUND

Fifty million individuals worldwide are currently living with Alzheimer's disease and related dementias (ADRD), a number expected to triple within 30 years as the global population ages.^1^ With few interventions that can effectively halt or delay disease progression,^2^ primary prevention of dementia has become a global goal.

After accumulating evidence that individual behaviors antedate lower ADRD risk (e.g., eating a Mediterranean diet, pursuing daily physical activity),^3^ attention has shifted to the spaces in which individual behaviors occur.^4^, ^5^, ^6^ The neighborhoods in which older adults live constrain and influence the social, recreational, and dietary choices they make on a daily basis, as well as determine the physical/chemical stressors to which they are exposed, including air and water pollutants, noise, heat, and environmental disasters.^4^, ^5^, ^6^

In certain countries, dementia diagnoses and brain‐tissue pathology have been found to concentrate in neighborhoods with disadvantageous physical, social, and economic characteristics over and above the personal‐risk demographics of individuals living in those neighborhoods (i.e., age, sex, genetics, social class position).^7^, ^8^, ^9^, ^10^, ^11^, ^12^, ^13^ For example, net of individual characteristics, cohort and brain‐bank studies in the United States and United Kingdom have identified a greater risk among individuals living in the most socioeconomically disadvantaged neighborhoods relative to the least of: cognitive deficits, white matter damage, mild cognitive impairment, dementia, progression to dementia from non‐impairment, and Alzheimer disease–associated neuropathology.^7^, ^8^, ^9^, ^10^, ^11^, ^12^, ^13^ Similar investigations in Denmark and France have reported null findings.^14^, ^15^ Supplementary Table S1 provides full details on the extant empirical literature on neighborhood characteristics and dementia/dementia antecedents.

Collectively, this existing evidence suggests that neighborhood‐based interventions could offer a new avenue for primary dementia prevention^16^—one that may leverage existing resources outside the healthcare sector, influence whole communities at once, and operate without necessarily requiring individual behavior change. For example, interventions targeting individuals, such as neighborhood mobility voucher programs, have shown efficacy for reducing obesity and diabetes,^17^ while interventions targeting whole neighborhoods, such as vacant‐lot greening initiatives, have been found to influence area‐level trends in criminal behavior,^18^ diets,^19^ and mental health.^20^

The nature of neighborhood‐based risk for dementia remains undercharacterized, however.^16^ Critically, it is unclear when in the lifespan such risk emerges. The lion's share of the evidence (∼70% of published studies) on neighborhood‐ADRD associations arrives from studies of older adults who have either received diagnoses, donated their brains for *post mortem* study, or been observed over the last years of their life (Table S1). This limits causal inference and the identification of intervention opportunities for four reasons. First, it does not rule out reverse causation, whereby individuals in the long premorbid phase of ADRDs involuntarily migrate to less desirable neighborhoods as a consequence of illness (e.g., cognitive decline forces early outflow from the labor market). Second, it does not rule out the accumulation within disadvantaged neighborhoods of individuals at risk for ADRDs due to pre‐existing shared risk factors (e.g., low educational attainment earlier in life). Third, older adult samples are biased by healthy survivor bias. Fourth, it does not indicate when neighborhood interventions would need to be delivered to be effective.

To help address this limitation in the literature, we first investigated the full New Zealand (NZ) population to test the hypothesis that dementia diagnoses follow neighborhood socioeconomic (“disadvantage”) gradients across the entire country. Then, we turned to a deeply phenotyped population‐representative NZ‐birth cohort followed to age 45 (the Dunedin Study) to test the hypothesis that the geographic patterning of dementia is preceded by geographic gradients in dementia's antecedent risk factors and brain‐integrity differences by midlife, decades before ADRD endpoints typically emerge.

## 2 METHODS

### 2.1 Dementia in the NZ population: The integrated data infrastructure (NZ‐IDI)

The NZ‐IDI is a collection of deidentified, individually linked, whole‐of‐population administrative data sources that combine government records with individual health data from hospital, clinic, pharmacy, and laboratory records with nationwide coverage.^21^, ^22^, ^23^ The study population included the 1,695,447 individuals aged 31‐90 years who were born in NZ between 1929 and 1968 and resided in NZ for any time between July 1999 and June 2019. We selected this age range to capture the period of risk for both early‐onset and later‐onset dementia during the 20‐year period. We divided the population into age bands (born between 1929‐1938, 1939‐1948, 1949‐1958, and 1959‐1968). Ethical approval was obtained from the University of Auckland Human Participants Ethics Committee (Ref UAHPEC023995). Output data underwent confidentiality review by Statistics New Zealand‐Tatauranga Aotearoa. Informed consent was not obtained per rule 11(2)(c)(iii) of the NZ‐Health Information Privacy Code, which allows for anonymized health data research.

#### 2.1.1 Exposure measure: Index of deprivation

Residential neighborhood socioeconomic status (disadvantage) was assessed via the NZ‐Index of Deprivation (NZDep), an area‐based measure of socioeconomic disadvantage derived from nine Census variables capturing area‐level rates of unemployment, education, homeownership, and other domains (Supplementary Table S2 and Supplementary Appendix 1).^24^ NZDep ranks all NZ neighborhoods from least to most socioeconomically disadvantaged for all NZ‐Censuses from 1991 onward, at the smallest geographic unit reported by Statistics NZ (“statistical area 1” in 2018, encompassing ∼100‐200 residents). Neighborhood disadvantage scores were available in the NZ‐IDI beginning January 1, 2000, as decile scores, converted to quintiles for analysis. Neighborhood disadvantage score at baseline (first registered home address) was considered the primary exposure to limit the potential for reverse causation. Secondary sensitivity tests utilized mean disadvantage scores across up to 20 addresses during the 20‐year study period.

**TABLE 1:** Risk of dementia (hazard ratios, HR) by quintile of neighborhood disadvantage in New Zealand, the United Kingdom, and the United States.

	New Zealand (*N* = 1,408,812)	Pooled estimates from US and UK (*N* = 2,051,209)	England (*N* = 6,220)	California (*N* = 149,385)	Minnesota (*N* = 4,699)	Ohio (*N* = 253,421)	US veterans (*N* = 1,637,484)
	HR (95% CI)	Mean HR	HR (95% CI)				
Q2 vs. Q1	1.07 (1.02, 1.11)	1.14	1.44 (1.08, 1.90)	1.09 (1.05, 1.12)	1.00 (0.66, 1.50)	1.09 (1.04, 1.13)	1.08 (1.06, 110)
Q3 vs. Q1	1.13 (1.09, 1.18)	1.19	1.38 (1.00, 1.79)	1.15 (1.10, 1.20)	1.11 (0.75, 1.66)	1.17 (1.12, 1.23)	1.13 (1.11, 1.15)
Q4 vs. Q1	1.25 (1.20, 1.30)	1.28	1.39 (1.03, 1.87)	1.19 (1.14, 1.25)	1.28 (0.91, 1.85)	1.36 (1.29, 1.43)	1.17 (1.15, 1.19)
Q5 vs. Q1	1.43 (1.36, 1.49)	1.47	1.45 (1.06, 1.99)	1.19 (1.13, 1.26)	1.65 (1.09, 2.49)	1.76 (1.69, 1.84)	1.31 (1.29, 1.33)

*Note*: Q1 (reference group) = low disadvantage, Q5 = high disadvantage. New Zealand estimates are based on residential address at first contact and dementia diagnosis across a 20‐year study period. Estimates are adjusted for sex, age‐band, and ethnicity. Pooled estimates from the United States and United Kingdom represent the mean of effects reported in the least‐adjusted models from the five published studies analyzing neighborhood disadvantage quintiles and dementia diagnoses among epidemiological samples from England,^11^ California,^8^ Minnesota,^10^ and Ohio,^12^ and among US Veterans Health Administration patients.^13^ Details on these studies are provided in Supplementary Table S1. Similar studies with other samples that utilized tertiles and quartiles of disadvantage are not included here but are detailed in Supplementary Table S1.

#### 2.1.2 Outcome measure: Dementia diagnoses

We collected information about ADRD diagnoses using a previously published scheme.^23^, ^25^ Diagnoses were ascertained via ICD‐10 and ICD‐9 dementia codes in public hospitals and mortality records maintained by the NZ‐Ministry of Health as well as antidementia drug prescriptions in pharmaceutical records maintained by the NZ‐Pharmaceutical Management Agency. Hospital records were available from July 1999 to June 2019, mortality records from July 1999 to December 2018, and pharmaceutical records from November 2010 to June 2019. Although dementia in the community was likely under‐identified in our medical register‐based ascertainment scheme, it has been previously shown that cases identified through this scheme are classified accurately, with the majority also diagnosed in community‐based assessments.^23^ Further details on dementia ascertainment are in Supplementary Appendix 2.

**RESEARCH IN CONTEXT**

1. **Systematic review**: Previous studies have reported elevated risk of Alzheimer's disease and related dementias among individuals living in socioeconomically disadvantaged neighborhoods, in specific communities and contexts, regardless of individual sociodemographics and genetics.
2. **Interpretation**: This study reports the first whole‐of‐country analysis of neighborhood disadvantage and dementia risk alongside upstream investigation of dementia's antecedent risk factors and premorbid brain structure and cognitive function differences. Disadvantaged neighborhoods in New Zealand were found to have more residents with diagnosed dementia, and decades before dementia was diagnosed, residents tended to have more dementia risk factors and more of dementia's brain‐structure antecedents.
3. **Future directions**: Area‐based health disparities in dementing illnesses may reflect the concentration of risk factors and related brain structure differences decades before dementia symptoms typically emerge. Whether neighborhoods themselves causally influence dementia risk is unknown, nevertheless initiatives targeting risk at the area level may offer novel, scalable opportunities for primary disease prevention.

### 2.2 Dementia risk factors and antecedents in a New Zealand birth cohort: The Dunedin study

Birth‐cohort participants were members of the Dunedin Study. The full cohort comprises all individuals born between April 1972 and March 1973 in Dunedin, NZ, who were eligible based on residence in the province and who participated in the first assessment at age 3. The cohort represents the full range of socioeconomic status in the general population of NZ's South Island.^26^ On adult health, the cohort matches the NZ‐National Health and Nutrition Survey on key indicators (e.g., body mass index, smoking, physical activity, visits to a physician)^26^ and the NZ‐Census of citizens of the same age on educational attainment.^27^ The cohort is primarily white; 7.5% self‐identify as having Māori ancestry, which matches the ethnic distribution of the South Island. Assessments were carried out at birth and ages 3, 5, 7, 9, 11, 13, 15, 18, 21, 26, 32, 38, and, most recently, at age 45 years (data collection completed April 2019) when 94% (*N* = 938) of the cohort members still alive participated. Participants gave written informed consent, and Study protocols were approved by the NZ‐Health and Disability Ethics Committee.

#### 2.2.1 Exposure measure: New Zealand index of deprivation

Residential neighborhood disadvantage was again assessed via the NZDep, described above. Neighborhood disadvantage scores were available for Study waves at ages 26, 32, 38, and 45 years (from 1998 to 2019). A cumulative adult neighborhood disadvantage score was generated via confirmatory factor analysis producing a single unitary factor from the four Study waves via maximum likelihood estimation with robust standard errors in MPlus (Version 7.11), with factor loadings set to one for each age to equalize disadvantage contributions across time (χ^2^ = 95.607, *p* < 0.001, CFI = 0.834, TLI = 0.815, RMSEA = 0.096, SRMR = 0.092). Sensitivity tests utilizing simple mean‐disadvantage scores did not change the pattern of results.

*Outcome Set 1: Dementia risk‐factor index* included five indexes which are suitable for use in midlife (Supplementary Table S3):^3^

**FIGURE 1:**
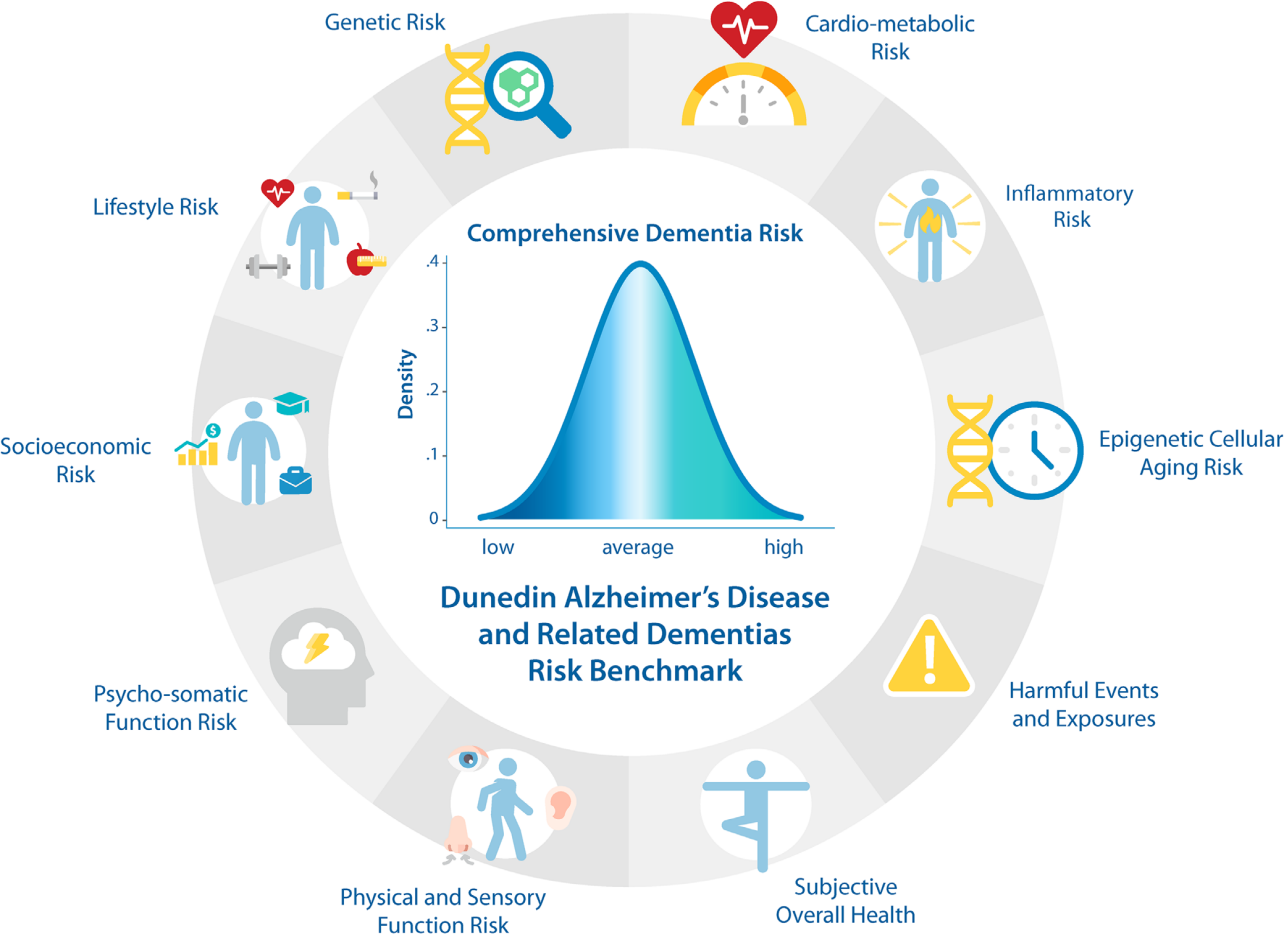
Schematic of the Dunedin Alzheimer's Disease and Related Dementias Risk Benchmark (DunedinARB). The comprehensive Dunedin Alzheimer's Disease and Related Dementias Risk Benchmark (DunedinARB) is comprised of 48 risk indicators grouped into 10 conceptually distinct domains. Genetic risk includes family history of dementia and apolipoprotein E (APOE) ε4 allele status. Lifestyle risk includes physical activity, diet, tobacco smoking, alcohol consumption, folic acid supplementation, and regular prophylactic non‐steroidal anti‐inflammatory drug (NSAID) use. Socioeconomic risk includes occupational and educational attainment. Psycho‐somatic Function risk includes chronic pain, history of migraine, history of depression, social isolation, sleep quality, neuroticism, and conscientiousness. Physical and Sensory Function risk includes balance, gait, hearing acuity, and subjective hearing function, objective and subjective vision function, and sense of smell. Cardio‐Metabolic Status risk includes hypertension, obesity, and diabetes status, total cholesterol, triglycerides, and retinal vascular health. Inflammatory risk includes C‐reactive protein (CRP), interleukin 6 (IL‐6), and soluble urokinase plasminogen activator receptor (suPAR)levels and history of rheumatoid arthritis. Epigenetic Cellular Aging risk includes four separate DNA methylation “aging” clocks (Horvath, Hannum, PhenoAge, and GrimAge). Harmful Events and Exposures risk includes childhood lead exposure, occupational exposure to neurotoxicants, and history of traumatic brain injury. Subjective Overall Health risk includes self, informant, and research‐worker ratings of Study member overall health. Figure from Reuben et al.^3^ Details on the individual risk factors and indicators are provided in Supplementary Table 4.

- The Cardiovascular Risk Factors, Aging, and Incidence of Dementia (CAIDE) index^28^;
- The LIfestyle for BRAin health (LIBRA) index^29^;
- The Australian National University Alzheimer's Disease Risk Index (ANU‐ADRI)^30^; and
- Modifiable risk factors selected by the Lancet Commission on Dementia (Lancet)^31^; and
- A comprehensive midlife index, the Dunedin ADRD Risk Benchmark (DunedinARB), comprised of 48 putative ADRD risk indicators organized into 10 conceptually distinct risk domains (Figure 1; Supplementary Table S4).^3^

These indexes are checklists that aggregate risk factors known to increase dementia risk because they are either robustly predictive of dementia (e.g., hearing impairment, a history of depression) or are believed to mechanistically precipitate or enhance disease processes (e.g., apolipoprotein E [APOE] genotype status, heart disease). Figure 1 and Supplementary Figure S1 present the risk factors captured in the DunedinARB and the four other indexes, respectively.

*Outcome Set 2: Brain structural and functional integrity* included seven midlife measures that are known antecedents of ADRD (Supplementary Table S5).^32^, ^33^, ^34^, ^35^, ^36^ These included four MRI‐measures of structural brain integrity (mean cortical thickness; bilateral hippocampal volume; total volume of white matter hyperintensities (WMH); and an MRI‐derived estimate of brain age^37^) and three measures of brain functional integrity (objective cognitive function assessed via cognitive tests; subjective cognitive function assessed via self‐ and informant‐report; and cognitive decline assessed as change in cognitive test scores from childhood).

### 2.3 Statistical analysis

The full project premise and analysis plan were preregistered and stored at https://tinyurl.com/5ytbax52. Findings were checked for reproducibility by an independent data analyst, who recreated the code based on the manuscript and applied it to a fresh dataset. This report follows STROBE reporting guidelines.^38^ Analyses conducted in SAS v7.1 and Stata v16.1.

#### 2.3.1 Analyses in the New Zealand‐IDI study population

Poisson regression models with relative risks (RRs) and Cox proportional hazard models with hazard ratios (HRs) (with censoring for out‐migration or death from causes other than dementia) were used to (1) estimate the association of neighborhood disadvantage with dementia per quintile increase in disadvantage, and (2) compare higher disadvantage quintiles against the lowest quintile. We used the neighborhood‐disadvantage score at individuals’ first registered residential address in the 20‐year study period (primary exposure) for both RRs and HRs and the mean disadvantage score across up to 20 addresses during the study period for RRs (sensitivity tests). Data were weighted for Poisson models based on time alive and in NZ to account for differences between individuals in observation time due to death or out‐migration. No measures were excluded at baseline.

Associations were estimated within the total study population with present neighborhood data (analytic sample), and by age band and sex. All models controlled for ethnicity (European, Māori, Pacific, and Asian ethnicity); models using the total population also controlled for sex and birth year. Per the confidentiality rules of Statistics‐NZ, reported frequencies/counts were randomly rounded to a base of three.

#### 2.3.2 Analyses in the New Zealand birth cohort

Ordinary least‐squares regression models were used to estimate the association of cumulative neighborhood disadvantage with the ADRD risk indexes and the brain structural and functional integrity measures, adjusted for sex. Second‐stage models were adjusted for sex and individual socioeconomic status at age 45 years (Supplementary Appendix 3).

## 3 RESULTS

### 3.1 Do dementia diagnoses follow neighborhood socioeconomic gradients in the New Zealand population?

We observed 1,695,447 individuals (full study population, 842,028 [49.7%] female; aged 31–70 years at baseline) who were born in NZ between 1929 and 1968 and resided in NZ for any period between July 1999 and June 2019; 231,567 (13.7%) were born between 1929 and 1938, 361,200 (21.3%) between 1939 and 1948, 504,372 (29.7%) between 1949 and 1958, and 598,308 (35.3%) between 1959 and 1968. During the 20‐year period, 36,753 individuals (2.2%) were identified as having dementia. Similar percentages of men and women and more older than younger individuals were identified as having dementia (Supplementary Table S6).

**FIGURE 2:**
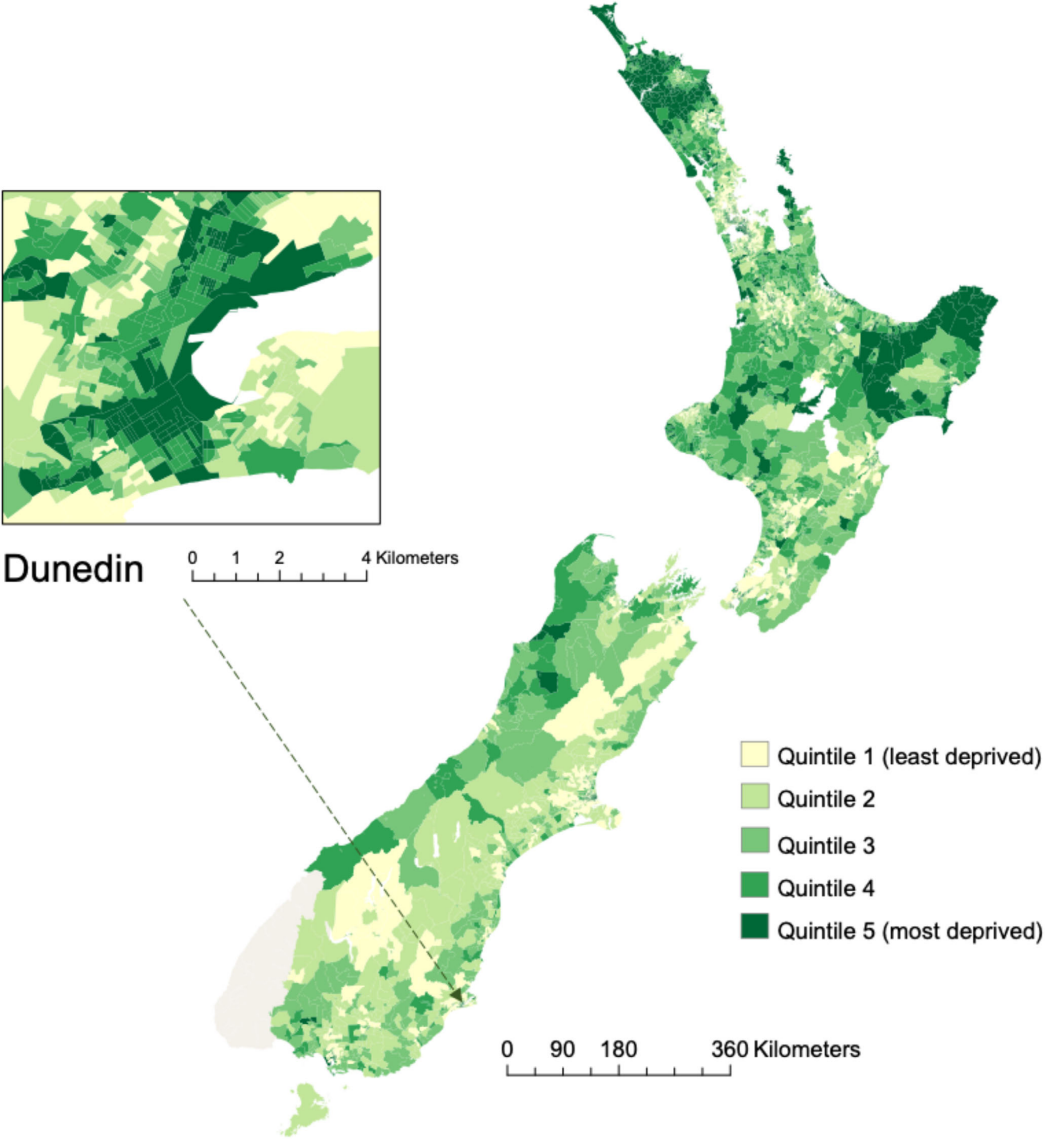
Geographic distribution of neighborhood socioeconomic disadvantage in New Zealand and the city of Dunedin, New Zealand (2018). The New Zealand Index of Deprivation ranks all populated small areas (encompassing approximately 100‐200 residents each on average) from least (quintile 1) to most (quintile 5) socioeconomically disadvantaged. Disadvantaged areas are distributed across both the South and North Island, with the North Island having a slightly larger share of the most disadvantaged areas. Unpopulated areas (Fiordland) pictured in gray.

**FIGURE 3:**
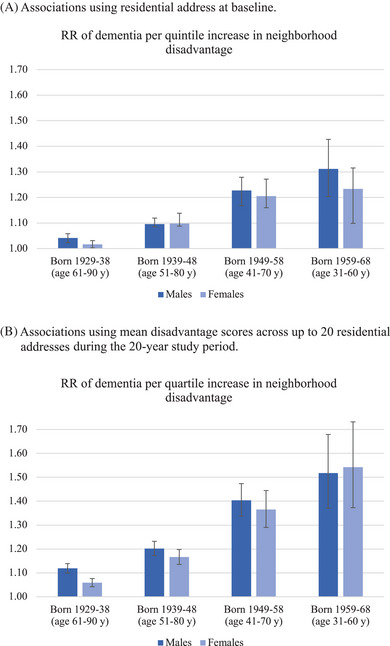
Neighborhood disadvantage associations with dementia diagnoses in the New Zealand study population, by sex and age cohort.

Figure 2 displays the geographic distribution of neighborhood disadvantages in NZ. Neighborhood disadvantage data were available for 1,408,812 individuals (the analytic sample, 83.1% of the study population; 652,581 [46.3%] female) (Supplementary Table S7). After adjustment for covariates, New Zealanders living in more disadvantaged neighborhoods at baseline were modestly more likely to develop dementia across the 20‐year observation period (RR per‐quintile‐increase in disadvantage = 1.06, 95% confidence interval [CI]: 1.05‐1.06; HR = 1.09, 95%CI: 1.08‐1.10). In general, neighborhood‐dementia associations were modestly greater among men than women and observably greater among more recently born, younger age‐bands. The youngest age‐band demonstrated risk associations 21.2% (for females) to 26.0% (for males) greater than the oldest age band (Figure 3, Panel A).

Neighborhood‐dementia associations increased modestly when all residential addresses across the observation period were taken into account during the 20‐year window (dementia RR per‐quintile‐increase in cumulative disadvantage = 1.13, 95%CI: 1.12‐1.14). Sex and age‐band differences followed the same general trend of higher risk associations among men and among more recently born age bands (Figure 3, Panel B).

Pairwise comparisons of higher disadvantage quintiles against the lowest quintile did not identify significant statistical‐effect thresholds: Neighborhood‐dementia associations increased linearly with increasing disadvantage (Table 1). Comparisons of NZ‐population neighborhood‐dementia HRs to those reported from specific samples in the United States and the United Kingdom using similar area‐level disadvantage indexes are presented in Table 1; the NZ population findings are similar these pooled HR estimates from previous studies.

### 3.2 Are neighborhood‐disadvantage gradients in dementia diagnoses preceded by gradients in dementia risk factors and structural and functional brain antecedents by midlife?

The midlife Dunedin Study included the 938 (49.5% female) Study members who attended the age‐45 assessment wave (94.1% of the original birth‐cohort members alive at age 45; see Supplementary Appendix 4 for wave‐45 attrition analysis). Data on cumulative adult neighborhood disadvantage (ages 26 to 45) were available for 907 Study members (96.7% of wave‐45). The 31 Study members lacking neighborhood data had higher cognitive ability (mean of 110.07 IQ points vs. 99.65 IQ points for Study members with neighborhood data; t = 3.83, *p* < 0.001) and higher individual‐level socioeconomic status (mean of 4.26 on a 6‐point social‐class scale vs. 3.71 for Study members with neighborhood data; t = 2.04, *p* < 0.001), reflecting the tendency of Study members who immigrated outside of New Zealand and Australia to gain higher‐status professions, typically in the United States or United Kingdom.^39^ Cumulative neighborhood disadvantage was normally distributed in the cohort (Supplementary Figure S2).

**FIGURE 4:**
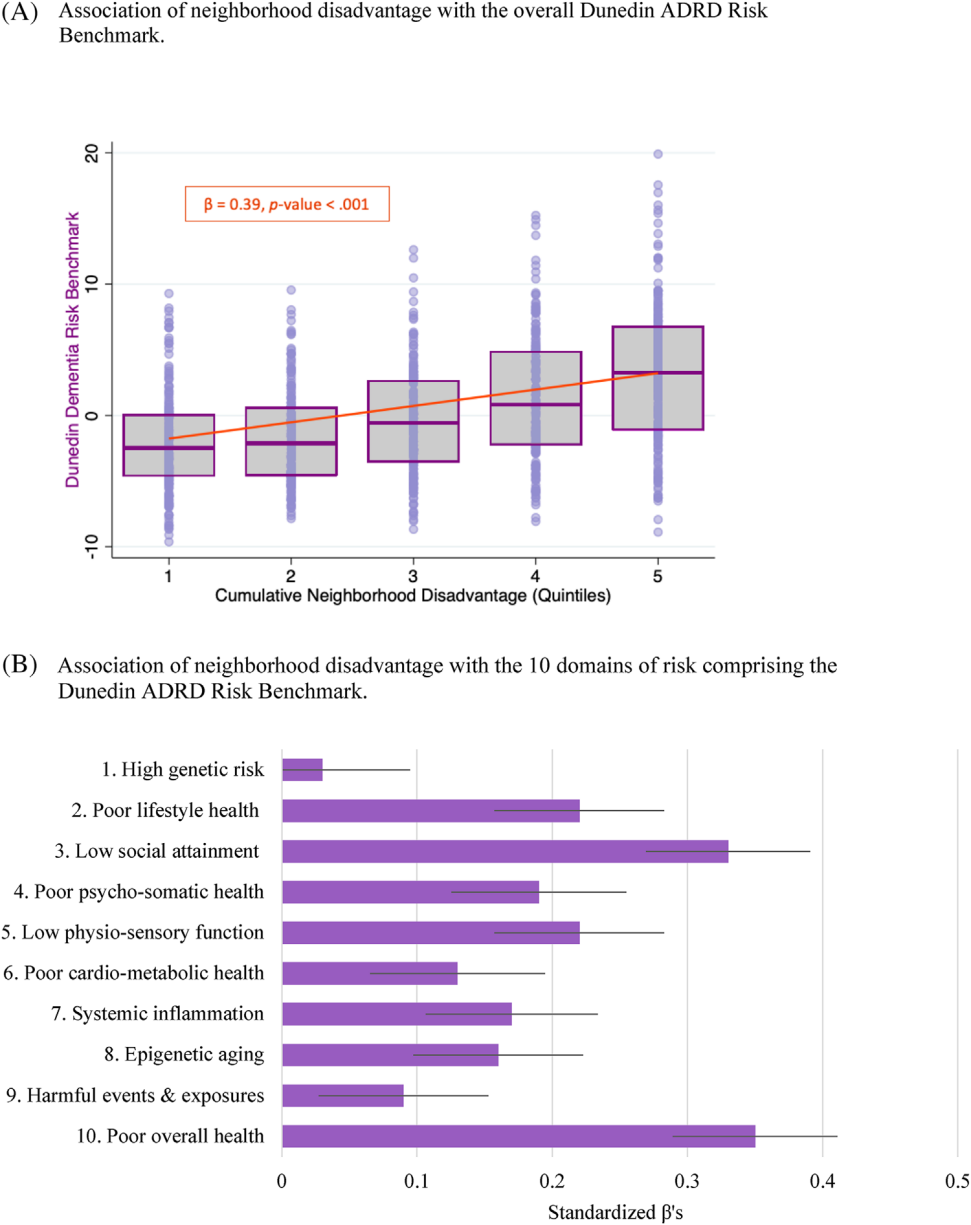
Association of cumulative adult neighborhood disadvantage with Alzheimer's disease and related dementias (ADRD) risk factors in the New Zealand Dunedin Study at age 45 years. Panel A presents box plot and underlying data points for Dunedin ADRD Risk Benchmark scores at each quintile of neighborhood disadvantage. Boxes display means and 25^th^ and 75^th^ percentile scores. Orange line presents the unadjusted trend for the association, with the association's standardized beta coefficient reported in the orange box above the trend line. Panel B: all *p* < 0.001 except for cardio‐metabolic risk (*p* = 0.005), harmful events and exposures risk (*p* = 0.009), and genetic risk (*p* = 0.504). Dark lines present 95% confidence intervals.

By age 45 years, Study members living in more disadvantaged neighborhoods across adulthood demonstrated significantly greater accumulation of midlife risk factors for later ADRD (Table 2) (β’s of 0.31 to 0.39 for associations with the CAIDE, LIBRA, Lancet, and ANU‐ADRI risk indexes and the DunedinARB, *p*’s < 0.001). Figure 4 (Panel A) presents the distribution of DunedinARB scores at each quintile of neighborhood disadvantage. Risk‐index‐associations remained significant after adjustment for individual‐level socioeconomic position (Table 2, column 2), although statistical effect sizes were attenuated (adjusted β’s of 0.19 to 0.24, *p*’s < 0.001). Examination of the 10 domains of risk comprising the DunedinARB indicated that no single risk domain accounted for the geographic patterning of dementia risk‐index scores (Figure 4, Panel B). All domains of risk were elevated among Study members who had lived in disadvantaged neighborhoods across adulthood (β’s of 0.08 to 0.35, *p*’s < 0.05), with the exception of genetic risk (β = 0.02, *p* = 0.504) (Supplementary Table S8). This pattern of associations remained after adjustment for individual‐level socioeconomic status, although effect sizes were attenuated, one to non‐significance (“harmful events and exposures” risk) (Supplementary Table S8). Overall, Study members from the most disadvantaged neighborhoods (5^th^ quintile) exhibited DunedinARB scores a full standard deviation higher (1.06 SD, 95%CI: 0.86, 1.27; t = 10.377, *p* < 0.001) than members from the least disadvantaged neighborhoods (1^st^ quintile).

**TABLE 2:** Association of cumulative adult neighborhood socioeconomic disadvantage with ADRD risk factors and brain integrity antecedents by midlife in the Dunedin Study cohort.

	Adjusted for sex	Adjusted for sex and individual social class position
	β (95%CI)	
Midlife ADRD risk indexes		
CAIDE	0.31^***^ (0.25, 0.37)	0.20^***^ (0.14, 0.26)
LIBRA	0.32^***^ (0.26, 0.39)	0.19^***^ (0.13, 0.24)
Lancet	0.35^***^ (0.29, 0.41)	0.24^***^ (0.18, 0.30)
ANU‐ADRI	0.34^***^ (0.28, 0.40)	0.21^***^ (0.15, 0.27)
DunedinARB	0.39^***^ (0.33, 0.45)	0.24^***^ (0.18, 0.29)

	**b (95%CI)**	
Midlife brain integrity measures		
Gray matter (mean cortical thickness, mm)	−0.01^**^ (−0.02, −0.004)	−0.007^*^ (−0.01, −0.0006)
Hippocampal volume (mm^3^)	−0.29 (−0.79, 0.22)	0.06 (−0.45, 0.58)
White matter (total white matter hyperintensities volume, log mm^3^)	0.07^*^ (0.02, 0.13)	0.04 (−0.02, 0.10)
BrainAGE (years)	1.20^***^ (0.67, 1.73)	0.86^***^ (0.30, 1.43)
Objective cognitive function (full‐scale IQ)	−3.59^***^ (−4.54, −2.64)	−1.10^*^ (−1.97, −0.23)
Subjective cognitive function (everyday cognitive complaints, z‐score)	0.14^***^ (0.09, 0.19)	0.08^**^ (0.03, 0.14)
IQ decline from childhood (residualized cognitive change, IQ scale)	−1.42^***^ (−2.03, −0.81)	−0.65^*^ (−1.27, −0.02)

*Note*: Cumulative adult neighborhood disadvantage spans ages 26–45 years. Fully adjusted associations include the covariates of sex and individual‐level socioeconomic status at age 45 years. Associations with the midlife ADRD indexes are presented as standardized regression beta coefficients. Associations with the midlife brain integrity measures represent unit change in outcomes per one standard deviation increase in neighborhood disadvantage.

Abbreviations: ADRD, Alzheimer's disease and related dementias; ANU‐ADRI, Australian National University Alzheimer's Disease Risk Index; CAIDE, Cardiovascular Risk Factors, Aging, and Incidence of Dementia; LIBRA, LIfestyle for BRAin health.

^*^ *p* < 0.05.

^**^ *p* < 0.01.

^***^ *p* < 0.001.

At age 45 years, Study members living in more disadvantaged neighborhoods across adulthood also demonstrated lower structural brain integrity (Table 2): each standard deviation increase in neighborhood disadvantage was associated with a 0.01 mm thinner average cortex (95%CI: −0.02, −0.004, *p* = 0.002, β = −0.11), a 0.07‐log‐mm^3^ greater white matter hyperintensities (WMH) volume (95%CI: 0.02, 0.13), *p* = 0.012, β = 0.09), and an additional 1.20‐years older brain age (95%CI: 0.67, 1.73, *p* < 0.001, β = 0.15). There was no statistically significant association between neighborhood disadvantage and bilateral hippocampal volume (b = −0.29 mm^3^, 95%CI: −0.79, 0.22, *p* = 0.263, β = −0.03). This overall pattern of associations remained after adjustment for individual‐level socioeconomic status, although effect sizes were attenuated, one to non‐significance (WMH) (Table 2). Overall, Study members from the most disadvantaged neighborhoods (5^th^ quintile) exhibited mean brain ages 2.98‐years older (95%CI: 1.18, 4.79; t = 3.255, *p* = 0.001) than members from the least (1^st^ quintile).

At age 45 years, Study members living in more disadvantaged neighborhoods across adulthood also demonstrated lower cognitive function: each standard deviation increase in neighborhood disadvantage was associated with an additional 3.59‐point lower full‐scale IQ score (95%CI: −4.54, −2.64, *p* < 0.001, β = −0.24), a 0.14‐standard deviation higher score on self‐ and informant‐reported everyday cognitive problems (95%CI: 0.09, 0.19, *p* < 0.001, β = 0.18), and an additional 1.42‐point decline in full‐scale IQ score from childhood to midlife (95%CI: −2.03, −0.81, *p* < 0.001, β = −0.15). This pattern of associations remained after adjustment for individual‐level socioeconomic status, although effect sizes were attenuated (Table 2).

## 4 DISCUSSION

This study produced three findings. First, dementia diagnoses were found to follow linear neighborhood socioeconomic gradients in the NZ population. Risk of dementia was over 20% greater in the most disadvantaged neighborhoods compared to the least. This replicates past reports of similar gradients in specific communities in the United States and United Kingdom (Supplemental‐Table S1) but is, to our knowledge, the first whole‐of‐country report (*N* = 1.4 million). Observed differences in dementia‐risk by age‐band suggest that neighborhood‐dementia associations may be driven primarily by neighborhoods predicting (or, if causal, elevating) risk of an “early” disease course (clinical symptoms emerging before age 75). It is also possible that at older ages the relative influence of neighborhoods on risk weakens as other factors play larger proportionate roles. Or, in older‐adult cohorts, individuals susceptible to neighborhood‐level dementia causes may have already died, whereas younger cohorts include individuals who represent the full variety of dementia causes.

Second, neighborhood gradients in dementia diagnoses were found to be preceded, in a population‐representative NZ‐birth cohort, by the geographic aggregation of dementia risk factors as early as age 45. Regardless of the risk‐index utilized, midlife risk for later dementia was significantly higher among individuals living in more disadvantaged neighborhoods across adulthood. Such risk included all putative ADRD risk factors, from lifestyle risks such as tobacco and alcohol use, to metabolic, psychiatric, and sensory dysfunction (e.g., high cholesterol, history of depression, poor hearing). The notable exception was genetic risk. *APOE*‐ε4 status and a family history of dementia were entirely unassociated with neighborhood disadvantage in the cohort—a finding matched in older adult studies, where neighborhood‐dementia association estimates tend to be unchanged by adjustment for genetic risk.^7^, ^10^, ^40^, ^41^ This suggests that neighborhood‐dementia gradients are not generated by the selective migration of individuals at genetic risk, at least by midlife. Robustness of neighborhood‐risk associations after adjustment for individual socioeconomic status indicates that geographic concentration of ADRD risk factors occurs over and above potential aggregation due solely to the social class make‐up of individuals living within neighborhoods.

Third, the concentration of ADRD risk factors among birth‐cohort members living in disadvantaged neighborhoods was accompanied by lower scores on midlife measures of brain structural and functional integrity that are known antecedents of ADRD. Individuals who had spent their adulthoods in disadvantaged neighborhoods demonstrated, by age 45, thinner cortices, greater burdens of white matter hyperintensities, and older looking brains, although they did not demonstrate specifically smaller hippocampi. They also experienced lower tested cognitive performance, greater problems with everyday cognitive function (such as misplacing eyeglasses or having difficulty following conversations), and greater longitudinal cognitive decline. Robustness of associations to adjustment for individual socioeconomic status indicates, once again, that geographic concentration of ADRD antecedents occurs over and above the social class make‐up of individuals living within neighborhoods. While statistical effect‐sizes were modest (as expected with MRI measures),^42^ these findings hold potential implications for the prevention of disease at the population‐level. Findings of neighborhood‐brain‐integrity associations as early as age 45 suggest that neighborhood‐based interventions could begin early, well before ADRD symptoms typically emerge.

There are two avenues for such neighborhood‐based dementia interventions. The first, and most straightforward (an individual‐level intervention), would be to facilitate neighborhood mobility for individuals already identified as high‐risk for dementia (e.g., because of APOE status, family history, a high risk‐index score, or early disease symptoms). Such an initiative would be similar to the UK and US governments’ respective Housing Benefit^43^ and Housing Choice Voucher^44^ programs, which financially assist some citizens (typically elderly or low‐income) in securing private‐market housing. As proof of the power of such programs, in a randomized‐controlled trial of a voucher program facilitating mobility to lower‐poverty neighborhoods, the US Department of Housing and Urban Development's Moving to Opportunity Study identified significant reductions in the prevalence of dementia risk factors of obesity and diabetes after several years among women who received the treatment.^17^ It has now been nearly three decades since enrollment in that study began—potentially enough time to identify whether the prevalence of dementia or dementia antecedents are also lower among members of the treatment group. Notably, individual‐level neighborhood interventions could be effective while remaining entirely agnostic about any causal mechanisms that may or may not underlie the neighborhood‐dementia association.

The second avenue for neighborhood‐based dementia interventions would be to improve neighborhood conditions directly (a neighborhood‐level intervention), targeting conditions known to influence the development of the dementia risk factors identified in the current study as aggregating in disadvantaged neighborhoods (Supplementary Table S8). Efforts to target such conditions, extensively investigated in the urban design literature,^16^ include altering and enhancing pedestrian infrastructure, natural and recreational amenities, the availability of fresh produce, and the accessibility of healthcare services. They also include efforts to reduce nervous system stressors such as heat, noise, and air pollution through altered fuel, air quality, and zoning codes and standards, cooling centers, tree plantings, road‐diets, and road‐space rationing. Investigation of just one such initiative, a randomized‐controlled trial of vacant‐lot greening in Philadelphia, found that low‐cost improvements to neighborhood spaces result in significant reductions in neighborhood‐level crime^18^ and poor mental health.^20^, ^45^ Although mental disorder is a risk factor for dementia,^23^ to the best of our knowledge, such neighborhood‐level interventions have yet to be investigated in relation to dementia or dementia antecedents.

This study has limitations. First, it is observational and cannot establish causation. Second, we did not have access to individual‐level socioeconomic information for the entire NZ‐IDI population; effect estimates would likely be attenuated after adjustment for individual‐SES, although the attenuation observed in the Dunedin Study suggests associations would remain significant. Third, administrative‐register data are known to undercount dementia cases. Fourth, brain‐integrity measures in the Dunedin Study were largely, although not exclusively, cross‐sectional. Continued follow‐up will be required to further estimate ADRD risk (e.g., using plasma biomarkers) as well as to document neighborhood‐associations with longitudinal declines in brain structure. Fifth, neighborhood disadvantage was only assessed across adulthood, leaving open the possibility that neighborhood‐brain‐integrity associations could be present earlier in the lifespan.

## 5 CONCLUSIONS

Risk of dementia is greater in more disadvantaged neighborhoods, reflecting a generally linear neighborhood‐socioeconomic‐gradient that has been identified in multiple communities and now, for the first time, at a country‐wide population level. While the mechanisms underlying this phenomenon remain poorly characterized, findings reported here suggest that neighborhood‐dementia gradients arise, in part, through the geographic concentration of dementia risk factors as early as midlife—decades before clinical disease endpoints typically emerge. Moreover, even at the relatively young age of 45, neighborhood disadvantage was accompanied, in members of the Dunedin Study, by modest, premorbid differences in brain structural and functional integrity that are known antecedents of dementia. This new evidence suggests that neighborhood‐based interventions could offer significant, scalable avenues for primary dementia prevention, ones that could come online long before disease endpoints emerge.

## CONFLICTS OF INTEREST STATEMENT

The authors declare no conflicts of interest. Author disclosures are available in the supporting information.

## CONSENT STATEMENT

For analyzes in the NZ‐IDI, informed consent was not obtained per rule 11(2)(c)(iii) of the NZ‐Health Information Privacy Code, which allows for anonymized health data research. Participants in the Dunedin Study birth cohort gave written informed consent, and Study protocols were approved by the NZ‐Health and Disability Ethics Committee.

## Supporting information

Supporting information

Supporting information

## Data Availability

Whole‐of‐country results for New Zealand are not official statistics. They have been created for research purposes from the Integrated Data Infrastructure, which is carefully managed by Statistics New Zealand. For more information about the Integrated Data Infrastructure, and to learn about dataset access, please visit https://www.stats.govt. nz/integrated‐data/. Statistics New Zealand approved the use of the Integrated Data Infrastructure for this project (ref MAA2019‐35). The Dunedin Study datasets reported in the current article are available on request by qualified scientists. Requests require a concept paper describing the purpose of data access, ethical approval at the applicant's university and provision for secure data access (https://moffittcaspi.trinity.duke.edu/research‐topics/dunedin). We offer secure access on the Duke, Otago, and King's College London campuses.
