# Supplementary material for: Dementia, dementia's risk factors and premorbid brain structure are concentrated in disadvantaged areas: National register and birth‐cohort geographic analyses
Source: Alzheimers Dement. 2024 Mar 14;20(5):3167–78. doi: 10.1002/alz.13727 (PMC11095428; doi:10.1002/alz.13727)
Supplement: Supplementary file 1 — Supporting information [file ALZ-20-3167-s001.docx]

**Supplement**

For manuscript entitled, “Dementia, dementia’s risk factors and premorbid brain structure are concentrated in disadvantaged areas: national register and birth-cohort geographic analyses.”

List of materials

[**Table S1.** Current literature on the association of neighborhood disadvantage with Alzheimer’s disease and related dementias pathology. 2](#_Toc153280430)

[**Table S2.** Census variables in the New Zealand Index of Deprivation (NZDep) by year. 7](#_Toc153280431)

[**Appendix 1.** Further details on the NZDep and data linkage process. 8](#_Toc153280432)

[**Appendix 2.** Ascertainment of Dementia in the NZ-IDI. 9](#_Toc153280433)

[**Table S3.** Assignment of risk points and weighting for each risk factor in the four external ADRD risk indexes. 10](#_Toc153280434)

[**Table S4.** Description of the risk indicators included in the Dunedin ADRD Risk Benchmark and assignment of risk points. 16](#_Toc153280435)

[**Figure S1.** Dementia risk factors selected by each of the four external risk indexes. 28](#_Toc153280436)

[**Table S5.** Description of the midlife brain integrity measures. 29](#_Toc153280437)

[**Appendix 3.** Ascertainment of individual-level socioeconomic status in the Dunedin Study. 31](#_Toc153280438)

[**Table S6.** Prevalence of dementia in the NZ-IDI. 32](#_Toc153280439)

[**Table S7.** Distribution of neighborhood disadvantage at first residential address in the NZ-IDI. 33](#_Toc153280440)

[**Appendix 4**. Age-45 Data Collection Wave attrition analysis. 34](#_Toc153280441)

[**Figure S2.** The distribution of neighborhood disadvantage in the Dunedin Cohort, from ages 26 to 45. 37](#_Toc153280442)

[**Table S8.** Association of neighborhood disadvantage with the 10 domains of risk comprising the Dunedin ADRD Risk Benchmark 40](#_Toc153280443)

[**References** 41](#_Toc153280444)

# **Table S1.** Current literature on the association of neighborhood disadvantage with Alzheimer’s disease and related dementias pathology.

| **First Author** | **Year** | **Sample** | **Country (State)** | **Age** | **N** | **Exposure Measure** | **Outcome Measure** | **Finding** |
| --- | --- | --- | --- | --- | --- | --- | --- | --- |
| Aneshensel^1^ | 2011 | Health and Retirement Survey (HRS) | United States | Mean: 59.35 [±3.23 SD]  Range: 54-65 | 4,525 | Neighborhood disadvantage score based on 1990 Census records | Cognitive status via Telephone Interview for Cognitive Status (TICS) | More deprived neighborhoods were associated with lower cognitive functioning. |
| Becerril^2^ | 2023 | Northeast Ohio Cohort for Atherosclerotic Risk Estimation (NEOCARE) Learning Health Registry | United States (OH) | Mean: 66  Range: ≥ 60 | 253,421 | Area Deprivation Index (ADI) | Dementia diagnosis via International Classification of Disease codes | Most- versus least-disadvantaged ADI quintile was associated with a greater risk of developing dementia (HR = 1.76). |
| Cadar^3^ | 2018 | English Longitudinal Study of Ageing | England | Median: 73.2 IQR: 68.1-78.3 | 6,220 | Index of Multiple Deprivation (IMD) | Dementia diagnosis via informant-reported physician diagnosis or a score above 3.38 on the Informant Questionnaire on Cognitive Decline in the Elderly | Most- versus least-disadvantaged quintile was associated with a greater risk of developing dementia (HR = 1.68). |
| Chamberlain^4^ | 2022 | Rochester Epidemiology Project | United States  (MN) | Median: 49  Range: ≥ 20 | 197,578 | Area Deprivation Index (ADI) | Diagnoses of chronic conditions via International Classification of Diseases | Most- versus least-deprived quintile was associated with more dementia diagnoses (OR = 1.36). |
| Clarke^5^ | 2015 | Chicago Health and Aging Project (CHAP) | United States (IL) | Mean: 74.1 [±7.4 SD]  Range: ≥ 65 | 6,518 | Neighborhood quality characteristics relevant to cognitive function | Cognitive tests via East Boston Memory Test, symbol digit test, and Mini Mental State  Examination (MMSE) | Higher quality neighborhoods were associated with slower rates of cognitive decline. 8% of variation in cognitive function attributable to neighborhoods. |
| Dintica^6^ | 2023 | US Veterans Health Administration | United States | Mean: 68.6  [±7.7 SD]  Range: ≥ 55 | 1,637, 484 | Area Deprivation Index (ADI) | Dementia diagnosis via International Classification of Diseases and International Statistical Classification of Diseases and Related Health Problems codes | Veterans residing in more disadvantaged neighborhoods had an increased risk of dementia in models adjusted for demographic characteristics and comorbid conditions. Most- versus least-disadvantaged quintile was associated with a greater risk of developing dementia (HR = 1.22). |
| George^7^ | 2020 | Atherosclerosis Risk in Communities Cohort | United States | Mean: 54 [±5.6 SD] | 12,599 | Life-Course Neighborhood Socioeconomic Status (LC-SES) | Diagnosis of dementia or cognitive impairment via MMSE, Clinical Dementia Rating Scale, telephone interviews, MRI, and hospital and death certificate codes | Individual LC-SES predicts dementia but neighborhood LC-SES does not. A standard deviation greater individual LC-SES was associated with a 14% (Whites) and 21% (Blacks) lower risk of dementia. |
| Hamilton^8^ | 2021 | Brains for Dementia Research Cohort | United Kingdom | Mean: 84.8  Range: ≥ 60 | 846 | Index of Multiple Deprivation (IMD) | Post-mortem neuropathological assessment via plaque staining, and antemortem Clinical Dementia Rating assessment | Most- versus least-deprived quintile had significantly higher neurofibrillary tangle (OR = 1.81), neuritic plaque staging (OR = 2.09), and increased cerebral amyloid angiopathy (OR = 2.55). |
| Hunt^9^ | 2020 | Wisconsin Registry for Alzheimer’s Prevention and Wisconsin Alzheimer’s Disease Research Center cohort | United States (WI) | Mean: 63.9  [±8.1 SD]  Range: 44.4-90.4 | 893 | Area Deprivation Index (ADI) | Cerebral and hippocampal volume via MRI | Living in the most-disadvantaged quintile was associated with 4.1% lower hippocampal volume and 2.0% lower brain tissue volume compared to least-disadvantaged quintile. |
| Hunt^10^ | 2021 | Wisconsin Registry for Alzheimer’s Prevention and Wisconsin Alzheimer’s Disease Research Center cohort | United States (WI) | Mean: 59.8  [±7.2 SD]  Range: 41-85 | 601 | Area Deprivation Index (ADI) | Cortical thickness changes via MRI and cognitive tests via Preclinical Alzheimer’s Disease Cognitive Composite, Trail-Making Test (part B), Rey Auditory Verbal Learning Test, and Story Memory Delayed Recall | Living in the most-disadvantaged quintile was associated with significantly accelerated degeneration in Alzheimer signature regions (β [SE] = −0.02 [0.01], p = 0.004) and cognitive decline. |
| Kim^11^ | 2023 | Danish refugees | Denmark | Mean: 53.8  [±10.6 SD]  Range: ≥ 40 | 9,854 | Neighborhood deprivation based on principle component analysis of census SES variables. | Dementia diagnosis via International Classification of Disease codes | Neighborhood disadvantage was not associated with dementia risk in any group. [Null finding]. |
| Kuchibhatla^12^ | 2020 | Health and Retirement Study (HRS) | United States | Mean: 67.5  [±3.4 SD]  Median: 66.0 | 8,198 | Neighborhood Socioeconomic Status (NSES) | Cognitive class categorizations via Telephone Interview for Cognitive Status (TICS-m) and diagnoses for cardiovascular or cerebrovascular risk factors | Participants in the highest NSES quartile had 57% higher odds of being in the highest cognitive class compared to those in the lowest NSES quartile. |
| Marsh^13^ | 2021 | NYU Alzheimer’s Disease Research Center | United States (NY) | Mean: 72.8 [±7.4 SD] | 313 | Area Deprivation Index (ADI) | Cognitive Decline using the Brief Cognitive Rating Scale (BCRS) | ADI was a significant predictor of subjective cognitive decline even when race and ethnicity were included as covariates (p < 0.05), suggesting independent influence of neighborhood deprivation. |
| McCann^14^ | 2018 | Trinity, Ulster, & Department of Agriculture (TUDA) cohort | Ireland and United Kingdom | Mean: 74  [±8.3 SD]  Range: ≥ 60 | 5,186 | Area Deprivation via 2011 Pobal HP Deprivation Index for Small Areas in the Republic of Ireland and 2010 Northern Ireland Multiple Deprivation Measure | Mini-Mental Status Examination (MMSE) | Most- versus least-deprived quintile was associated with significantly higher risk of cognitive dysfunction indicated by an MMSE <25 (OR = 1.4). |
| Merkel^15^ | 2022 | Wisconsin Registry for Alzheimer’s Prevention and Wisconsin Alzheimer’s Disease Research Center cohort | United States (WI) | Mean: 66.59  [±7.95 SD] | 804 | Area Deprivation Index (ADI) | White matter hyperintensity volume was measured via the lesion prediction algorithm from the Lesion Segmentation Toolbox | The association between neighborhood-level disadvantage and white matter hyperintensity volume was not statistically significant. [Null] |
| Meyer^16^ | 2018 | UC Davis Alzheimer’s Disease Center (ADC) | United States (CA) | Mean: 74.4  [±7.33 SD]  Range: 45-93 | 480 | Neighborhood Socioeconomic Status (NSES) | Clinical evaluations via Clinical Dementia Rating and diagnoses, and cognitive testing via Spanish and English Neuropsychological Assessment Scales (SENAS) subtests for executive function, semantic memory, and episodic memory | At baseline, NSES was associated with semantic memory but not executive function or episodic memory. NSES was not associated with cognitive change longitudinally. |
| Meyer^17^ | 2023 | University of California, Davis Alzheimer’s Disease Research Center (ADRC). | United States (CA) | Mean: 75.1 [±7.0 SD] | 327 | Neighborhood Socioeconomic Status (NSES) based on six census variables | Cognitive Decline via the Spanish and English Neuropsychological Assessment Scales (SENAS) | Segregation and neighborhood SES were differentially associated with cognition depending on participant race. |
| Mobley^18^ | 2022 | Northern California integrated health care delivery system | United States (CA) | Mean: 71.6  [±7.74 SD]  Range: 60-89 (baseline) | 167,488 | Area Deprivation Index (ADI) | Dementia incidence via International Classification of Diseases diagnostic codes | Most- versus least-disadvantaged quintile was associated with dementia within non-Latino Whites (HR = 1.09) but not Asian Americans (HR = 1.01). |
| Ouvrard^19^ | 2017 | Personnes âgées QUID Cohort (PAQUID) | France | Mean: 74.94 [±6.62 SD]  Range: ≥ 65 | 3,431 | FDep99 (1999 deprivation index) | Dementia diagnoses including Alzheimer Disease, vascular dementia, fronto-temporal dementia, Lewy Body disease, and Parkinson dementia. | No increased risk of dementia was found for those living in communities with a high index of deprivation. [Null] |
| Pase^20^ | 2022 | Healthy Brain Project | Australia | Mean: 56.1  [±7.2 SD]  Range: 40-70 | 4,656 | Australian Bureau of Statistics Index of Relative Socio-economic Advantage and Disadvantage (IRSAD) | Cardiovascular Risk Factors, Aging, and Incidence of Dementia (CAIDE) and Cogstate Brief Battery | Each decile increase in IRSAD was associated with lower CAIDE dementia risk scores (β [SE] = −0.070 [0.019], p = .004) and better memory performance. |
| Powell^21^ | 2020 | Alzheimer Disease Research Center brain banks | United States (WI/CA) | Mean: 80.3  [±9.5 SD]  Range: ≥ 65 | 447 | Area Deprivation Index (ADI) | Alzheimer disease (AD) neuropathology as defined by the presence of either diffuse plaques or neuritic plaques | Each decile increase in neighborhood disadvantage was associated with an 8.1% increase in odds of AD neuropathology. The most disadvantaged decile was associated with a 2.18 increased odds of AD neuropathology. |
| Powell^22^ | 2022 | Alzheimer Disease Research Center brain banks | United States (WI/CA) | Mean: 80.5  [±9.1 SD]  Range: ≥ 65 | 428 | Area Deprivation Index (ADI) | Alzheimer disease–associated neurofibrillary pathology via neurofibrillary tangle B scores, per NIA and Alzheimer’s Association neuropathological guidelines | Each decile increase in neighborhood disadvantage was associated with a 5% greater odds of a higher tangle B score. The most disadvantaged decile had 56% increased odds of higher tangle B score. |
| Rosso^23^ | 2016 | Cardiovascular Health Study (CHS) | United States (NC/CA/MD/PA) | Mean: 74.8  Range: ≥ 65 | 3,595 | Neighborhood Socioeconomic Status (NSES) | Modified Mini-Mental State Examination (3MS), digit symbol substitution test (DSST), and white matter hyperintensities (WMH) | Higher NSES was associated with higher 3MS scores at baseline, but not with 3MS changes over time. NSES was marginally associated with DSST and not associated with WMH. |
| Sheffield^24^ | 2009 | Hispanic Established Populations for Epidemiologic Studies of the Elderly cohort | United States | Mean: 73.28  [±6.5 SD]  Range: ≥ 65 | 3,050 | Neighborhood economic advantage and neighborhood social disadvantage | Mini-Mental State Examination (MMSE) | Residents of the lowest quartile neighborhoods of economic advantage showed significantly higher odds of cognitive decline (OR = 1.75). |
| Shih^25^ | 2011 | Women’s Health Initiative Memory Study | United States | Mean: 70.2  [±3.9 SD]  Range: 65-81 | 6,137 | Neighborhood Socioeconomic Status (NSES) | Modified Mini-Mental State Examination (3MSE) | Each NSES unit increase was associated with a 0.0235 standard deviation higher 3MSE score. |
| Tan^26^ | 2023 | United Kingdom Biobank | United Kingdom | Mean: 54.76  [±7.47 SD]  Range: 40-69 | 19,638 | Townsend Deprivation Index | Hippocampus volume, regional cortical thickness, white matter hyperintensities, and cognition via fluid intelligence assessment | Participants with low SES demonstrated the smallest hippocampal volumes, greatest white matter hyperintensities volume, and poorer cognition if they resided in high deprivation neighborhoods; negative associations were reduced among those residing in low deprivation neighborhoods. |
| Thierry^27^ | 2021 | Health and Retirement Study (HRS) | United States | Mean: 74.18  Range: ≥ 65 | 8,023 | Perceived safety, cleanliness, and social cohesion via psychosocial questionnaire | Telephone Interview Cognitive Screen (TICS), Center for Epidemiologic Studies Depression scale (CES-D), and physical health risk factors | For Black participants, poor perceived neighborhood quality was associated with worse cognitive function among those with more education. For white participants, worse neighborhood characteristics correlated with poorer cognitive function among those with less education. Among Mexican participants, perceived neighborhood uncleanliness was associated with lower cognitive function among those with less education. |
| Vassilaki^28^ | 2022 | Mayo Clinic Study of Aging (MCSA) | United States | Mean: 72.9  [±10.5 SD]  Range: ≥ 50 | 4,699 | Area Deprivation Index (ADI) | Diagnosis of mild cognitive impairment (MCI) or dementia via DSM-IV and cognitive performance assessments via Short Test of Mental Status and neuropsychological tests | Higher ADI was associated with higher MCI odds (OR = 1.08) at baseline. In cognitively unimpaired participants, each decile increase in ADI was associated with a 1.06 higher risk of progression to dementia over 10 years of observation. |
| Wight^29^ | 2006 | Study of Assets and Health Dynamics Among the Oldest Old (AHEAD) | United States | Mean: 77.15  [±5.69 SD]  Range: ≥ 70 | 3,442 | US Census tract data specifically for educational attainment at the neighborhood level | Telephone Interview Cognitive Screen (TICS), Center for Epidemiologic Studies Depression scale (CES-D) | Older adults residing in low-education areas fare worse cognitively than those living in high-education areas (including effects caused by their own educational attainment). This association remains robust after adjustment for contextual-level median household income. |

# **Table S2.** Census variables in the New Zealand Index of Deprivation (NZDep) by year.

| **Deprivation domain** |  | **NZDep 2001** | **NZDep 2006** | **NZDep 2013** | **NZDep 2018** |
| --- | --- | --- | --- | --- | --- |
| *Telecommunication access* |  | Residents without telephone access |  | Residents under age 65 without access to the Internet at home | Residents without access to the Internet at home |
| *Public benefit receipt* |  | Residents aged 18-64 receiving a means-tested benefit |  |  |  |
| *Income* |  | Residents living in equivalised* households with income below an income threshold |  |  |  |
| *Employment* |  | Residents aged 18-59 years who are unemployed | Residents aged 18-64 years who are unemployed |  |  |
| *Education* |  | Residents aged 18-59 years without any qualifications | Residents aged 18-64 years without any qualifications |  |  |
| *Housing stability* |  | Residents not living in their own home |  |  |  |
| *Family structure* |  | Residents under age 60 living in a single-parent family | Residents under age 65 living in a single-parent family |  |  |
| *Housing structure* |  | Residents living in equivalised* households below a bedroom occupancy threshold |  |  |  |
| *Transportation/Living Conditions* |  | Residents without access to a car |  |  | Residents living in dwellings that are always damp and/or have mold greater than A4 size |

# **Appendix 1.** Further details on the NZDep and data linkage process.

The New Zealand Index of Deprivation (NZDep) has been generated for all New Zealand Censuses, from 1991 onward, at the smallest geographic unit for which statistical data is reported by Statistics New Zealand – New Zealand’s national statistics government agency. In 2018 “statistical area 1,” encompassing approximately 100-200 residents and no more than 500, replaced the historical smallest unit, the “meshblock,” which encompassed approximately 30-60 dwellings and no more than 120 dwellings.^30^

The NZDep ranks all small areas of New Zealand on measures of socioeconomic disadvantage derived from Census variables capturing area-level rates of unemployment, education, homeownership, and other domains (described in Supplementary-Table S2). NZDep scores are reported as raw ranks and as national deciles. In the NZ-IDI and Dunedin Birth Cohort, NZDep decile scores were generated for each known residential address based on the statistical area in which that address was based, as identified using address geocoding software that converts text-based addresses into geographic latitude/longitude coordinates which are then aligned via geospatial software with the known geographic boundaries of each statistical area. These NZDep decile scores are then linked to individual-level information using PIN codes.

Neighborhood disadvantage scores were available in the NZ-IDI beginning January 1^st^, 2000 and tracked all study population members residing in New Zealand for any time until the end of the study observation (June 2019). NZ-IDI analyses were weighted based on time alive and in New Zealand to account for differences between individuals in observation time due to death or out-migration

As it is common for New Zealand adults to spend significant portions of time living abroad, mainly in Australia, NZDep data was supplemented in the Dunedin Cohort by matched neighborhood disadvantage scores for individuals living in Australia for at least 9 months in a given assessment year, using the Australian Index of Relative Socioeconomic Advantage and Disadvantage, an area-based measure of disadvantage derived from 25 Census variables, calculated at “statistical areas level 1,” encompassing approximately 400 residents.^31^

# **Appendix 2.** Ascertainment of Dementia in the NZ-IDI.

Alzheimer’s disease and related dementias (ADRD) were ascertained using a previously-published scheme.^32,33^ We collected information about dementias using (a) International Classification of Diseases, 10th Revision (ICD-10) and corresponding ICD-9 dementia codes in public-hospital records, (b) ICD-10 and corresponding ICD-9 dementia codes in mortality records, and (c) anti-dementia drug prescriptions in pharmaceutical records. In order to obtain consistency across diagnostic schemes, corresponding diagnoses in the ICD-9 were ascertained using mapped codes provided by the New Zealand Ministry of Health. Mapping code is hosted at the following website: https://moffittcaspi.trinity.duke.edu/researchtopics/ statistical-code

ICD-10 and corresponding ICD-9 codes used to ascertain dementia from public-hospital and mortality records:

| **ICD-10** | **ICD-9** |
| --- | --- |
| F00, F00.0, F00.1, F00.2, F00.9, F01, F01.0,  F01.1, F01.2, F01.3, F01.8, F01.9, F02, F02.0, F02.1, F02.2, F02.3, F02.4, F02.8, F03, F05.1, F10.7, F13.7, F18.7, F19.7, G30, G30.0, G30.1, G30.8, G30.9, G31.0, G31.1, G31.3 | 290.0, 290.3, 290.8, 290.9, 290.10, 290.11, 290.12, 290.13, 290.20, 290.21, 290.40, 290.41, 290.42, 290.43, 291.2, 292.82, 294.1, 331.0, 331.1, 331.2 |

Anti-dementia drugs (and corresponding formulation IDs) used to ascertain dementia from pharmaceutical records:

- Donepezil (392325, 392326)
- Rivastigmine (403725, 403726)

Although dementia in the community was likely under-identified in our medical register–based ascertainment scheme, cases were classified accurately: 83.1% of cases diagnosed with dementia in medical registers were also diagnosed in community-based assessments. (eAppendix 3 in the Supplement of Richmond-Rakerd et al., 2022).^32^

*Note*. The drugs Galantamine and Memantine are also approved for use in New Zealand to treat dementia symptoms. However, we considered only the drugs listed above as these were the drugs included in the published dementia ascertainment scheme (developed from NZ Integrated Data Infrastructure health records) that we followed for the current report.^32^

# **Table S3.** Assignment of risk points and weighting for each risk factor in the four external ADRD risk indexes.

| **1**. **The** **CAIDE risk index**^34^ | | | | |
| --- | --- | --- | --- | --- |
|  | **Indicator** | **Level** | **Risk Points** |  |
|  | Age |  |  |  |
|  |  | <47 years old | 0 |  |
|  |  | 47-53 years old | 3 |  |
|  |  | >53 years old | 5 |  |
|  | Education |  |  |  |
|  |  | ≥10 years | 0 |  |
|  |  | 7-9 years | 3 |  |
|  |  | 0-6 years | 4 |  |
|  | Sex |  |  |  |
|  |  | Female | 0 |  |
|  |  | Male | 1 |  |
|  | Hypertension |  |  |  |
|  |  | SBP ≤ 140 mmHg | 0 |  |
|  |  | SBP >140 mmHg | 2 |  |
|  | Obesity (BMI) |  |  |  |
|  |  | ≤30 kg/m2 BMI | 0 |  |
|  |  | >30 kg/m2 BMI | 2 |  |
|  | Total cholesterol | |  |  |
|  |  | ≤6.5 mmol/l total cholesterol | 0 |  |
|  |  | >6.5 mmol/l cholesterol | 1 |  |
|  | Physical activity | |  |  |
|  |  | Active: physical activity at least twice a week, lasting at least 20–30 min each time, and causing sweating and breathlessness. | 0 |  |
|  |  | Inactive | 1 |  |
|  | APOE ε4 allele status |  |  |  |
|  |  | Non-carrier | 0 |  |
|  |  | Carrier of at least 1 ε4 allele | 2 |  |
|  |  |  |  |  |
|  |  | **Potential Total Points Range** | 0 to 18 |  |
|  |  | **Dunedin Study Total Points Range** | 0 to 13 |  |

| **2. The LIBRA risk index**^35^ | | |  |
| --- | --- | --- | --- |
|  | **Indicator** | **Level** | **Risk Points** |
|  | Hypertension |  |  |
|  |  | Not hypertensive | 0 |
|  |  | SBP ≥ 140 mmHg or DBP ≥ 90 mmHg | 1.6 |
|  | Obesity (BMI) |  |  |
|  |  | <30 kg/m2 BMI | 0 |
|  |  | ≥30 kg/m2 BMI | 1.6 |
|  | High cholesterol |  |  |
|  |  | <6.5 mmol/l total cholesterol | 0 |
|  |  | ≥6.5 mmol/l total cholesterol | 1.4 |
|  | Diabetes |  |  |
|  |  | Not diagnosed | 0 |
|  |  | Diagnosed | 1.3 |
|  | Coronary heart disease | |  |
|  |  | No disease | 0 |
|  |  | Myocardial infarction, angina, ischemic heart disease, or atrial fibrillation reported | 1 |
|  | Chronic kidney disease | |  |
|  |  | No disease | 0 |
|  |  | Chronic nephritis, chronic renal failure, and proteinuria reported | 1.1 |
|  | Physical inactivity | |  |
|  |  | Active: At least 20-30 minutes of daily or 2-3x weekly physical activity causing breathlessness and sweating. | 0 |
|  |  | Inactive | 1.1 |
|  | Low/moderate alcohol intake | |  |
|  |  | Consumes alcohol once every 2 months or less | -1 |
|  |  | Consumes alcohol once a month or more / Does not drink | 0 |
|  | Smoking |  |  |
|  |  | Never smoker | 0 |
|  |  | Ever smoker | 1.5 |
|  | Depression |  |  |
|  |  | Did not report feeling somewhat or more "hopeless" on questionnaire | 0 |
|  |  | Reported feeling somewhat or more "hopeless" on questionnaire | 2.1 |
|  | Healthy diet |  |  |
|  |  | Low adherence to a healthy diet (≤8 on a 17 point scale) | 0 |
|  |  | high adherence to a healthy diet (>8 on a 17 point scale) | -1.7 |
|  | Cognitive and social engagement^a†^ | |  |
|  |  | Self-reported engagement in intellectual and social activities | 0 |
|  |  | Self-reported engagement in intellectual and social activities | -3.2 |
|  |  |  |  |
|  |  | **Potential Total Points Range** | -5.9 to 12.7 |
|  |  | **Dunedin Study Total Points Range** | -2.7 to 12.7 |

| **3. The Lancet Commission**^36^ **risk factor list^b^** | | | |  |
| --- | --- | --- | --- | --- |
|  | **Indicator** | **Level** | **Risk Points** | |
|  | Education |  |  | |
|  |  | “More education”  (Operationalized as: high school graduate or above) | 0 | |
|  |  | “Less education”  (Operationalized as: left school without certification or high school degree) | 1.6 | |
|  | Hearing loss |  |  | |
|  |  | No hearing loss or hearing lose with use of a hearing aid | 0 | |
|  |  | Hearing loss without use of hearing aid | 1.9 | |
|  | Traumatic Head Injury |  |  | |
|  |  | No documented history of traumatic head injury | 0 | |
|  |  | History of traumatic head injury | 1.8 | |
|  | Hypertension | |  | |
|  |  | Not hypertensive | 0 | |
|  |  | SBP ≥ 140 mmHg or DBP ≥ 90 mmHg | 1.6 | |
|  | Excessive alcohol consumption | |  | |
|  |  | < 21 units alcohol consumed per week | 0 | |
|  |  | ≥ 21 units consumed per week | 1.2 | |
|  | Obesity (BMI) |  |  | |
|  |  | <30 kg/m2 BMI | 0 | |
|  |  | ≥30 kg/m2 BMI | 1.6 | |
|  | Smoking |  |  | |
|  |  | Non-smoker by midlife | 0 | |
|  |  | Current smoker by midlife | 1.6 | |
|  | Depression |  |  | |
|  |  | Never received a diagnosis by midlife | 0 | |
|  |  | Diagnosed episode by midlife | 1.9 | |
|  | Social isolation | |  | |
|  |  | Not socially isolated  (Operationalized as ≤1 SD above the cohort mean on a scale assessing loneliness) | 0 | |
|  |  | Socially isolated  (Operationalized as >1 SD above the cohort mean on a scale assessing loneliness) | 1.6 | |
|  | Physical activity | |  | |
|  |  | Active | 0 | |
|  |  | Inactive | 1.4 | |
|  | Diabetes |  |  | |
|  |  | No diagnosis | 0 | |
|  |  | Diagnosed | 1.5 | |
|  | Air pollution exposure | |  | |
|  |  | Low exposure | 0 | |
|  |  | High exposure^c^ | 1.1 | |
|  |  |  |  | |
|  |  | **Potential Total Points Range** | 0 to 18 | |
|  |  | **Dunedin Study Total Points Range** | 0 to 18 | |

| **4. The ANU-ADRI risk index**^37^ | | | |
| --- | --- | --- | --- |
|  | **Indicator** | **Level** | **Risk Points** |
|  | Age for males | |  |
|  |  | <65 years | 0 |
|  |  | ≥65 | 1 to 38 points |
|  | Age for females | |  |
|  |  | <65 years | 0 |
|  |  | ≥65 | 1 to 41 points |
|  | Education |  |  |
|  |  | > 11 years | 0 |
|  |  | 8-11 years | 3 |
|  |  | <8 years | 6 |
|  | Weight status if age <60 | |  |
|  |  | Reported as normal | 0 |
|  |  | Reported as overweight | 2 |
|  |  | Reported as obese | 5 |
|  | Diabetes |  |  |
|  |  | No diabetes reported | 0 |
|  |  | Diabetes reported | 3 |
|  | Symptoms of Depression | |  |
|  |  | CES-D score ≤ 16 | 0 |
|  |  | CES-D score > 16 | 2 |
|  | High cholesterol | |  |
|  |  | <6.2 mmol/l total cholesterol | 0 |
|  |  | ≥6.2 mmol/l total cholesterol | 3 |
|  | Traumatic Brain Injury |  |  |
|  |  | No history of TBI | 0 |
|  |  | Positive history of TBI | 4 |
|  | Smoking |  |  |
|  |  | Never smoker | 0 |
|  |  | Ever smoker | 1 |
|  |  | Current smoker | 4 |
|  | Alcohol intake | |  |
|  |  | No alcohol consumed | 0 |
|  |  | Light to moderate consumption | -3 |
|  |  | Heavy consumption | 0 |
|  | Social engagement^e^ | |  |
|  |  | Highest scores in sample | 0 |
|  |  | Lowest scores in sample | 6 |
|  |  | Low to medium scores in sample | 4 |
|  |  | Medium to high scores in sample | 1 |
|  | Physical activity | |  |
|  |  | High IPAQ score | 0 |
|  |  | Moderate IPAQ score | -2 |
|  |  | Low IPAQ score | -3 |
|  | Cognitive activity^f†^ | |  |
|  |  | Lowest cogntive activity questionnaire scores | 0 |
|  |  | Middle scores | -7 |
|  |  | Highest scores | -6 |
|  | Fish intake |  |  |
|  |  | <.25 servings per week | 0 |
|  |  | .25-2 servings per week | -3 |
|  |  | 2-4 servings per week | -4 |
|  |  | >4 servings per week | -5 |
|  | Pesticide exposure | |  |
|  |  | Never | 0 |
|  |  | Ever | 2 |
|  |  |  |  |
|  |  | **Potential Total Points Range** | -18 to 35 |
|  |  | **Dunedin Study Total Points Range** | -10 to 23 |

*Note*. BMI = Body Mass Index; SBP = Systolic Blood Pressure; DBP = Diastolic Blood Pressure; TBI = Traumatic Brain Injury. CES-D = Center for Epidemiologic Studies Depression scale (CES-D). IPAQ = the International Physical Activity Questionnaire

^†^This measure was not available in the Dunedin Cohort.

^a^Self-reported engagement in intellectual and social activities in the last 12 months (e.g., read the newspaper on a daily basis, have a hobby, take a holiday, using the internet, being a member of any organizations).

^b^At the time of study there were no previous publications assessing risk prediction using the Lancet Commission risk factor list. Lancet Index cut-points were consequently developed based on existing clinical thresholds (e.g., for weight status and hypertension), the presence of categorical conditions (e.g., depression diagnosis), cut-points used by other indices (e.g., for physical activity), and extreme scores on continuous measures relative to cohort peers (e.g., for social isolation). Risk scores were assigned based on the Lancet Commission’s published relative risk for dementia scores for each risk indicator (Table 1, Column 1 in Livingston et al., 2020).^36^

^c^High air pollution exposure was operationalized as residence for one year or more in a poor air quality city (i.e., top 500 worst air quality cities as ranked by IQAir, www.iqair.com) or a country with average annual outdoor ambient air quality below World Health Organization standards.

^e^Social engagement in the ANU-ADRI development was measured via a composite score encompassing marital status, size and quality of social networks, level of social activities and living arrangements. It was operationalized in the Dunedin cohort as reverse coded high (≥ 6), medium to high (2.1 to 5), low to medium (0.1 to 2), and low (0) scores on the UCLA Loneliness Scale.^38^

^f^ANU-ADRI utilized a modified form of the Rush Memory and Aging Study cognitive activity questionnaire and assigned risk points based on the distribution of scores within the test sample.

# **Table S4.** Description of the risk indicators included in the Dunedin ADRD Risk Benchmark and assignment of risk points.

| Risk domain^a^ | Risk indicator | Description | Risk point assignment^b^ |
| --- | --- | --- | --- |
| Genetic risk^39,40^ | Family history of dementia | Study members reported at age 45 on family history of dementia. (N=925 with present data before imputation, 99% of the analytic sample). | Risk points were assigned (0 [92.8% of cohort]; 1 [7.1%]; or 2 [0.1%]) based on number of parents with diagnosed dementia. These match the prevalence rates of dementia in the wider New Zealand population^33^ and other similar populations characterized by a large proportion of individuals of European descent (e.g., the United States).^41^ |
|  | APOE ε4 allele status | Number of APOE ε4 alleles was assessed. APOE protein iosoforms E2/E3/E4 were derived from phased haplotypes of SNPs rs7412 and rs429358 assayed on a genome-wide array, Infinium OmniExpress-12 v1.1 BeadChip array (Illumina Inc., San Diego, California). (N=848 with present data before imputation, 90% of the analytic sample). | Risk points were assigned (0 [69.7%]; 1 [27.2%]; or 2 [3.1%]) based on the number of ε4 alleles present. The overall APOE E4 allele frequency was 17.0%, which is typical for people of European descent, somewhat smaller than what is typical for people of African descent, and somewhat larger than what is typical for people of Asian descent.^42^ |
|  |  |  |  |
| Lifestyle risk^43–48^ | Physical activity | Study members reported at age 45 on hours per week of leisure-time physical activity at moderate or greater levels of intensity. Cohort range: 0 to 22, mean(SD) = 2.82 (3.26). (N=908 with present data before imputation, 97% of the analytic sample). | Risk points were assigned based on World Health Organization guidelines for adults aged 18-64 years^49^: -1 = >3hrs weekly (33.8%); 0 = 1-3hrs weekly (31.0%); 1 = <1hr weekly (35.2%). |
|  | Diet | Study members reported at age 45 on their weekly diet. A scale assessing adherence to a Mediterranean-style diet was comprised of Study member report about their typical consumption per week (none, <1, 1-2 times, 3-4 times, most days, or daily) of meat, fish, nuts, beans, fruit, vegetables, sugary drinks, and extra virgin olive oil. Higher scores indicate closer adherence to a healthy diet (higher in fish, nuts, beans, fruit, vegetables, and olive oil, and lower in meat and sugary drinks). Resulting scale was z-scored, cohort range = -2.84 to 2.69; mean(SD) = 0(1). (N=900 with present data before imputation, 96% of the analytic sample). | Risk points were assigned as: -1 = >1SD above the mean (15.8%); 0 = within 1 SD of the mean inclusive (70.9%); 1 = < 1SD below the mean (13.3%). |
|  | Tobacco smoking | Study members reported at age 45 on daily tobacco smoking habits. (N=924 with present data before imputation, 99% of the analytic sample). | Risk points were assigned based on classification as non-smokers (0 [47.8% ]), former smokers (1 [30.3%]), and current smokers (2 [22.0%]). |
|  | Alcohol consumption | Study members reported at age 45 on weekly alcohol consumption habits. Consistent with published ADRD risk indices^50,51^, moderate drinkers were designated as lower risk that non-drinkers or heavy drinkers. (N=938 with present data before imputation, 100% of the analytic sample). | Risk points were assigned based on classification as light drinkers (-1 [40.5%]), non-drinkers (0 [7.3%]), and heavy drinkers (1 [52.2%]). Light drinkers consumed ≤ 7 or 14 drinks per week, respectively for females and males, and reported fewer than 6 occasions of binge drinking (5 or more drinks in a single occasion) in the past year. |
|  | Folic acid supplementation | Study members reported at age 45 on whether they regularly took folic acid or B complex supplementation. (N=938 with present data before imputation, 100% of the analytic sample). | Risk points were assigned as: -1 = Yes (1.8%); 0 = No (98.2%). |
|  | Regular prophylactic NSAID use | Study members reported at age 45 on whether they regularly took non-steroidal anti-inflammatory drugs (NSAIDs). (N=938 with present data before imputation, 100% of the analytic sample). | Risk points were assigned as -1 = Yes (20.8%); 0 = No (79.2% of cohort). |
|  |  |  |  |
| Socioeconomic risk^52–54^ | Occupational attainment | Study members reported at age 45 on their occupational attainment. Occupations were scored using the New Zealand Socioeconomic Index (NZSEI-06), which codes each occupation based on its associated education level and income in the NZ Census (score range, 10 [low status]-90 [high status]) and groups occupations into 6 status levels. Examples of occupations in the 6 groups include medical practitioner (NZSEI code 90; group 6), engineering professional (code 66; group 5), database administrator (code 59; group 4), personal assistant (code 44; group 3), office cashier (code 28; group 2), and fish filleter (code 23; group 1). Homemakers and others not working in the past year were assigned the status of their most recent occupation, as reported at age 38. Study members who had been out of the labor force since age 32 were assigned the status of their partner if they had a partner with whom they shared a household. (N=935 with present data before imputation, 99% of the analytic sample). | Risk points were assigned as: -1 = High status (groups 5 and 6) (33.2%); 0 = Middle status (groups 3 and 4) (46.2%); 1 = Low status (groups 1 and 2) (20.7%). |
|  | Educational attainment | Study member’s reported on their educational attainment by age 45^55^ and were grouped into ranked education levels of: 0 = no certifications (N = 138, 14.7%); 1 = school certification achieved (N = 135, 14.4%); 2 = high school graduate or equivalent (N = 376, 40.1%); and 3 = Bachelor’s degree or higher (N = 288, 30.7%). (N=937 with present data before imputation, 99% of the analytic sample). | Risk points were assigned as: -1 = Bachelor’s degree or higher (30.7%); 0 = high school graduate or equivalent / school certification (54.6%); 1 = no school certification (14.7%) |
|  |  |  |  |
| Psychological and somatic function risk^56–61^ | Pain interference with daily function | Study members reported at age 45 on pain interference with daily life (0=not at all, to 5=very much) via questions about the extent to which pain hinders engagement with social, cognitive, emotional, physical, and recreational activities. Cohort range: 0-20, mean(SD)=3.24(4.24). (N=908 with present data before imputation, 97% of the analytic sample). | Risk points were assigned as: 0 = ≤1 SD above the mean (84.8%); 1 = >1 SD above the mean (15.2%). |
|  | History of migraine | Study members reported at each assessment wave from age 26 to 45 about whether they had experienced frequent headaches lasting from 30 min to 7 days in the past year. Headache pain characteristics and symptoms were also assessed and headaches classified as either tensio-type or migraine.^62,63^ A count of phases with migraine headaches was created. (N=938 with present data before imputation, 100% of the analytic sample). | Risk points were assigned as: 0 = never met criteria for migraine in adulthood (75.4%); 1 = met criteria for migraine at at least one wave (24.6%). 47.2% of Study members who met criteria for migraine did so at multiple waves. |
|  | History of depression | Study members reported at each assessment wave from age 15 to 45 about about symptoms of Major Depressive Disorder over the past year. 50.5% of Study members met criteria for depression at at least one wave and 28.4% met criteria at multiple waves. (N=938 with present data before imputation, 100% of the analytic sample). | Risk points were assigned as: 0 = 1 or fewer episodes of depression across adulthood (71.6%); 1 = >1 episode of depression across adulthood (28.4%). |
|  | Loneliness / social isolation | Study members reported at age 45 on loneliness and social isolation via response (0=hardly ever, 1=some of the time, 2=often) to four items adapted from the UCLA Loneliness Scale^38^ (e.g., “How often do you feel you lack companionship?”; “How often do you feel isolated from others?”). Items were summed to create a loneliness / social isolation scale. Cohort range: 0-8, mean(SD)=1.21(1.77). (N=922 with present data before imputation, 98% of the analytic sample). | Risk points were assigned as: 0 = ≤1 SD above the mean (80.4%); 1 = >1 SD above the mean (19.6%). |
|  | Sleep quality | Study members reported at age 45 on sleep quality using the Pittsburgh Sleep Quality Index (PSQI).^64^ The PSQI consists of 18 self-report items relating to individuals’ sleep patterns and different forms of sleep impairment in the past month. These questions are used to derive scores for seven different components of sleep (subjective sleep quality, sleep latency, sleep duration, habitual sleep efficiency, sleep disturbances, use of sleep medication and daytime dysfunction), each scored from 0 to 3. These were summed to produce a global score ranging from 0 to 21, with higher scores reflecting worse sleep quality. Cohort range: 1-17, mean(SD)=6.24(2.41). (N=909 with present data before imputation, 97% of the analytic sample). | Risk points were assigned as: 0 = ≤1 SD above the mean (85.4%); 1 = >1 SD above the mean (14.6%). |
|  | Neuroticism & Conscientiousness | At the age 45 assessment, informants nominated by the Study members as people "who knew them well" were mailed questionnaires and asked to describe each Study member using a 25-item version of the Big Five Inventory, which measured the personality traits of Neuroticism and Conscientiousness.^65^ Items such as "Can be moody" and "Is emotionally stable, not easily upset" asssesed Neuroticism and items such as "Does a thorough job" and "Makes plans and follows through with them" assessed Conscientiousness. Resulting scales ranged from 0 to 10: cohort mean(SD) Neuroticism = 3.96(2.02) and Conscientiousness = 7.48(1.54). (N=883 with present data before imputation, 94% of the analytic sample). | Risk points for Neuroticism were assigned as: 0 = ≤1 SD above the mean (81.2%); 1 = >1 SD above the mean (18.8%). Risk points for Conscientiousness were assigned as: -1 = >1 SD above the mean (14.0%); 0 = ≤1 SD above the mean (86.0%). |
|  |  |  |  |
| Physical and Sensory function risk^66–71^ | Balance | Balance was measured at age 45 using the Unipedal Stance Test as the maximum time achieved across three trials of the test with eyes closed.^72^ Cohort range: 1-30, mean(SD)=14.58(9.72). (N=911 with present data before imputation, 97% of the analytic sample). | Risk points were assigned as: 0 = ≥1 SD below the mean (86.4%); 1 = <1 SD below the mean (13.6%). |
|  | Gait | Gait speed (meters per second) was assessed at age 45 with the 6-m-long GAITRite Electronic Walkway (CIR Systems, Inc) with 2-m acceleration and 2-m deceleration before and after the walkway, respectively. Gait speed was assessed under 3 walking conditions: usual gait speed (walk at normal pace from a standing start, measured as a mean of 2 walks) and 2 challenge paradigms, dual task gait speed (walk at normal pace while reciting alternate letters of the alphabet out loud, starting with the letter “A,” measured as a mean of 2 walks) and maximum gait speed (walk as fast as safely possible, measured as a mean of 3 walks). We calculated the mean of the 3 individual walk conditions to generate our primary measure of composite gait speed.^73^ Cohort range: 0.74-2.12, mean(SD)=1.41(0.19). (N=904 with present data before imputation, 96% of the analytic sample). | Risk points were assigned as: 0 = ≥1 SD below the mean (84.9 = <1 SD below the mean (15.1%). |
|  | Objective hearing function (hearing acuity) | Hearing thresholds were measured at age 45 by conducting pure-tone audiometry. In a sound-attenuating booth, pure-tones delivered in the following order of frequencies – 1000 Hz, 2000 Hz, 4000 Hz, 8000 Hz, 12500 Hz, and 500 Hz – were presented to the study members through headphones. Presentation intensity levels began at 40 decibels at hearing level (dB HL) for normal hearing study members, and 60 dB HL for hearing aid users. Study members used a response button to indicate whenever they heard a tone, and the lowest intensity level that elicited a response was identified as the hearing threshold for that frequency. A high PTA was calculated by averaging 8000 Hz and 12500 Hz. Results from the best ear are reported. Cohort range: -7.5 to 85, mean(SD)=22.12(14.75). | Risk points were assigned as: 0 = ≥1 SD below the mean (84.2%); 1 = <1 SD below the mean (15.8%). |
|  | Subjective hearing function | Study members reported at age 45 on hearing problems via responses to 3 items from the Speech, Spatial, and Qualities of Hearing Scale (SSQ12)^74^ (e.g., “Can you follow the conversation in a busy restaurant?”). Cohort range: 0-29, mean(SD)=7.86(5.12). (N=924 with present data before imputation, 99% of the analytic sample). | Risk points were assigned as: 0 = ≤1 SD above the mean (82.2%); 1 = >1 SD above the mean (17.8%). |
|  | Objective vision function | Contrast sensitivity was measured at age 45 using a Pelli-Robson chart administered by trained technicians. The chart presents 3 letters per line and the letters gradually fade from black to gray to white on a white background to determine the lowest level of “contrast” that the eye can detect. The resulting measure is a contrast sensitivity score function, reflecting a person’s best-corrected contrast detection threshold, the lowest contrast at which a pattern can be seen. Cohort range: 1.4-2.25, mean(SD)=2.00 (0.13). (N=904 with present data before imputation, 96% of the analytic sample). | Risk points were assigned as: 0 = ≥1 SD below the mean (96.1%); 1 = <1 SD below the mean (3.9%). |
|  | Subjective vision function | Study members reported on vision difficulties at age 45 via responses on the 10-item Vision Quality of Life Core Measure (VCM1) questionnaire^75^ (e.g., “How often has your eyesight stopped you from doing the things you wanted to do?”). Cohort range: 0-42, mean(SD)= 3.61 (4.70). (N=925 with present data before imputation, 99% of the analytic sample). | Risk points were assigned as: 0 = ≤1 SD above the mean (88.0%); 1 = >1 SD above the mean (12.0%). |
|  | Poor sense of smell | Study members reported at age 45 on poor sense of smell via response to the question, "Have you had problems with your sense of smell, such as not being able to smell things, or things not smelling the way they should" that had lasted for at least 3 months. (N=931 with present data before imputation, 100% of the analytic sample). | Risk points were assigned as: 0 = No (96.8%); 1 = Yes (3.2%). |
|  |  |  |  |
| Cardio-metabolic function risk^76–81^ | Blood pressure (hypertension) | Systolic and diastolic blood pressure were assessed at age 45 according to standard protocols with a BpTRU™ Vital Signs Monitor BPM 200. (N=906 with present data before imputation, 97% of the analytic sample). | Risk points were assigned based on classification as non-hypertensive (0 [82.0%]) or hypertensive (1 [18.0%]). Study members were considered hypertensive if they had systolic blood pressure 140 mm Hg or greater or diastolic blood pressure 90 mm Hg or greater. |
|  | BMI (weight status) | Height was measured at age 45 using a Seca 264 Wireless Stadiometer. Weight was measured at age 45 to the nearest 0.1 kg using calibrated scales. Individuals were weighed in light clothing. Body mass index (BMI) was calculated. Cohort range: 16.17-62.17, mean(SD)=28.45(5.77). (N=920 with present data before imputation, 98% of the analytic sample). | Risk points were assigned based on classification as non-obese (0 = <30BMI, 66.2%) and obese (1 = ≥30BMI, 33.8%). |
|  | Diabetes status | Whole blood glycated hemoglobin concentration (A1C) (expressed as a percentage of total hemoglobin) was measured at age 45 by ion exchange high performance liquid chromatography (Variant II: BioRad, Hercultes, Calif.), a method certified by the US National Glycohemoglobin Standardization Program (<http://www.ngsp.org/>). (N=876 with present data before imputation, 93% of the analytic sample). | Risk points were assigned based on classification as non-diabetic (0 = <6.5% A1C level, 97.7% of the cohort) and diabetic (1 = ≥6.5% A1C level, 2.3% of cohort). |
|  | Total cholesterol and triglycerides | Serum non-fasting total cholesterol and triglycerides levels were measured at age 45 by colorimetric assay on a Cobas c702 analyzer. (N=879 with present data before imputation, 94% of the analytic sample). | Risk points were assigned based on clinical thresholds for high cholesterol (1 = ≥6.5 mmol/L, 8.1%) and high triglyceridies (1 = >2.26 mmol/L, 33.2%). |
|  | Retinal vascular health | Vascular health was measured at age 45 through assessment of retinal venular vessel calibers. Digital fundus photographs were taken after 15 min of dark adaptation and graded at the SNEC Ocular Reading Centre, Singapore National Eye Centre and Measurements using trained graders following standardized protocols with higher intergrader reliability. Vessel calibers were recorded for arterioles and venules where they passed through a region located 0.50 to 2.00 disk diameters from the optic disk margin and summarized as central retinal artery equivalent (CRAE) and central retinal vein equivalent (CRVE).^82^ Cohort range -2.71 to 3.72, mean(SD) = 0.01(0.98). (N=890 with present data before imputation, 95% of the analytic sample). | Risk points were assigned as: 0 = ≥1 SD below the mean (84.1%); 1 = <1 SD below the mean (15.9%). |
|  |  |  |  |
| Inflammatory risk^83–85^ | CRP level | Serum C-reactive protein (mg/L) was measured at age 45 using particle-enhanced immunoturbidimetric assays on a Cobas c702 analyzer (Roche Diagnostics GmbH) following standard procedures. The lower detection limit of the assay was 0.3 mg/L. The intraassay and interassay CVs reported by the manufacturer were *r* = 0.28–1.34% and *r* = 2.51–5.70%, respectively. Values were log-transformed for analysis to account for positive skew. Cohort range 0-4.51, mean(SD) = 0.97(0.69). (N=879 with present data before imputation, 94% of the analytic sample). | Risk points were assigned as: 0 = ≤1 SD above the mean (85.8%); 1 = >1 SD above the mean (14.2%). |
|  | Il-6 level | Serum IL-6 (pg/mL) was measured at age 45 using an electrochemiluminescence immunoassay on a Cobas e 602 analyzer (Roche Diagnostics GmbH) following standard procedure. The lower detection limit of the assay was 1.5 pg/mL. The intraassay and interassay CVs reported by the manufacturer were 2.5–6.0% and 2.9–8.5%, respectively. Cohort range 0.40-28.47, mean(SD) = 2.18(2.54). (N=876 with present data before imputation, 93% of the analytic sample). | Risk points were assigned as: 0 = ≤1 SD above the mean (93.9%); 1 = >1 SD above the mean (6.1%). |
|  | SuPAR level | Plasma suPAR (ng/mL) was measured at age 45 with the suPARnostic AUTO Flex ELISA (ViroGates A/S, Birkerød, Denmark) according to manufacturer’s instructions. The detection limit of the assay was 0.1 ng/mL. The intraassay correlation of repeat measurements of the same sample was *r* = 0.98 and coefficient of variation (CV) = 2.4%, and the interassay correlation was *r* = 0.81 and CV = 12.8%. Cohort range 0.87-14.37, mean(SD) = 3.07(1.06). (N=875 with present data before imputation, 93% of the analytic sample). | Risk points were assigned as: 0 = ≤1 SD above the mean (89.8%); 1 = >1 SD above the mean (10.2%). |
|  | Rheumatoid arthritis status | Study members reported at age 45 on their history of rheumatoid arthritis during a general health screen. (N=926 with present data before imputation, 99% of the analytic sample). | Risk points were assigned as: 0 = No current arthritis diagnosis (98.2%); 1 = Current arthritis diagnosis (1.8%). |
|  |  |  |  |
| Cellular aging (DNA methylation clock) risk^86,87^ | 4 separate DNA methylation aging clocks | DNA methylation was measured at age 45 as CpG methylation Beta values derived from leukocyte DNA samples using the EPIC array (Illumina Inc., San Diego, California). Methylation values were transformed into four separate DNA methylation “aging” clocks: Horvath,^88^ Hannum,^89^ PhenoAge,^90^ and Grim.^91^ Horvath and Hannum represent first-generation clocks, trained on chronological age in diverse samples. PhenoAge and Grim represent second-generation clocks, trained on phenotypic biomarkers associated with aging (e.g., white blood cell count, albumin levels, etc.). All clocks were calculated using the online calculator found at <https://dnamage.genetics.ucla.edu/new>. ‘Normalization’ and ‘advanced analysis in blood’ options were selected, and data were anonymized prior to upload. From the results file, the corresponding epigenetic age calculations (DNAmAge, DNAmAgeHannum, DNAmPhenoAge, DNAmGrimAge) were extracted. (N=819 with present data before imputation, 87% of the analytic sample). | Risk points were assigned for each DNA methylation clock as: 0 = ≤1 SD above the mean; 1 = >1 SD above the mean.  Horvath: 16.4% at risk  Hannum: 15.4% at risk  PhenoAge: 15.1% at risk  Grim: 16.5% at risk |
|  |  |  |  |
| Harmful events and exposures^92–98^ | Early life lead exposure | Blood-lead level (ug/dL) was assessed at age 11 years. Approximately 30ml of venous blood was collected and whole blood samples analyzed through graphite furnace atomic absorption spectrophotometry. Details on the method of blood collection, storage, and analysis have been previously described.^99,100^ Cohort lead exposure matched that of other same-aged cohorts tested in the United States and United Kingdom.^100^ Cohort range: 4-31, mean(SD) = 10.85(3.61). (N=541 with present data before imputation, 58% of the analytic sample). | Risk points were assigned as: 0 = ≤1 SD above the mean (88.7%); 1 = >1 SD above the mean (11.3%). |
|  | Occupational exposure to pesticides | Study members reported at age 45 on their current occupation. (N=877 with present data before imputation, 93% of the analytic sample). | Risk points were assigned based on occupations that include exposure to pesticides, insecticides, fungicides, or timber preservatives (1 point, 9.9% of cohort) or not (0 points, 90.1 %). |
|  | History of traumatic brain injury | Study member history of traumatic head injury was assessed prospectively by asking at each assessment wave if they experienced a head injury requiring medical attention plus aftercare over the past year. Parents reported on head injury at assessment waves in childhood and adolescence and Study members self-reported at adult assessment waves. (N=938 with present data before imputation, 100% of the analytic sample). | Risk points were assigned as: 0 = no history of traumatic head injury by age 45 (88.5%); 1 = history of traumatic head injury (11.5%). |
|  |  |  |  |
| Subjective Overall Health^101,102^ | Self-rated health | Study members reported at age 45 on their subjective overall health in response to the question, "In general, would you say your health is: excellent, very good, good, fair, poor." Cohort range: 1-5, mean(SD) = 3.66(0.90). (N=931 with present data before imputation, 99% of the analytic sample). | Risk points were assigned as: -1 = very good to excellent (59.3%); 0 = good (31.7%); 1 = fair to poor (9.1%). |
|  | Informant-rated health | Informants who knew the Study members well rated Study member overall health (excellent, very good, good, fair, poor) on questionnaires mailed to them during the age 45 assessment. Cohort range: 1-5, mean(SD) = 3.79(0.89). (N=883 with present data before imputation, 94% of the analytic sample). | Risk points were assigned as: -1 = very good to excellent (67.3%); 0 = good (25.3%); 1 = fair to poor (7.5%). |
|  | Research worker-rated health | Study research workers reported on their subjective impressions of Study member overall health (excellent, very good, good, fair, poor) at the age 45 assessment. Staff ratings were obtained from four raters for each Study member: the cardiovascular nurses, the sensory technicians, the Study Director, and the Assessment Manager (who was in charge of informed consent and logistics on the in-Unit assessment day). Cohort range: 1-5, mean(SD) = 3.36(0.84). (N=934 with present data before imputation, 99% of the analytic sample). | Risk points were assigned as: -1 = very good to excellent (47.7%); 0 = good (39.1%); 1 = fair to poor (13.3%). |
|  |  |  |  |

*Note.* Indicators were present for 93-100% of the analytic sample for all indicators except DNA methylation clocks and childhood blood-lead level, which were available for only 87% and 58% of the cohort respectively; missing indicator values were imputed to create complete indicator data for the entire cohort present at the age-45 wave. Removing the lead-level indicator did not change the results.

^a^Representative empirical studies, meta-analyses, systematic reviews, and narrative reviews are cited to describe the evidence of associations of known or proposed risk factors within the 10 risk domains with ADRD. These are cited for expository purposes and are not meant to reflect all the evidence available on a given risk domain, factor, or indicator.

^b^Risk points were assigned after imputation to address missing data. Risk point assignments were not weighted as meta-analyses were not available for all risk indicators. The DunedinARB construction allowed each risk domain to contribute equally to the overall risk score.

# **Figure S1.** Dementia risk factors selected by each of the four external risk indexes.

# **Table S5.** Description of the midlife brain integrity measures.

|  |  | **Description** |
| --- | --- | --- |
| **Brain structural integrity measures** | | |
|  | **Grey Matter:** Mean Cortical Thickness, mm | At age 45, Study members completed a brain magnetic resonance imaging (MRI) protocol using a Siemens Skyra 3T scanner (Siemens Healthcare) equipped with a 64-channel head and neck coil.  Mean cortical thickness was derived from T1-weighted images using FreeSurfer Version 5.^103^  Analytic sample N = 861, M (SD) = 2.55 (0.09) mm |
|  | Hippocampal Volume, mm^3^ | Total bilateral volume of the hippocampus^104^, a region of the brain important for learning and memory, was measured using FreeSurfer’s automated segmentation (ASEG) algorithm.  Analytic sample N = 861, M (SD) = 86.45 (8.5) mm^3^ |
|  | **White Matter:**  White Matter Hyperintensities Volume, log mm^3^ | Total volume of white matter hyperintensities^105^ was measured using the Unidentified Bright Objects (UBO) algorithm applied to T2-weighted fluid attenuated inversion recovery images. White matter hyperintensities are bright spots on a T2 weighted MRI scan that are a sign of deterioration of white matter. The variable was log-transformed to produce an approximately normal distribution.  Analytic sample N = 852, M (SD) = 6.52 (0.80) log mm^3^ |
|  | **Age-related biomarker**:  BrainAGE, years | T1- and T2-weighted images were used to estimate brain age for each Study member. Specifically, we calculated a brain age gap estimate (brainAGE), which is the difference between each Study member’s actual chronological age at the time of scan and their predicted age from MRI data.^106^ Positive brainAGE values indicate a brain that looks morphologically “older” than expected based on chronological age..  Analytic sample N = 869, M (SD) = 0.00 (8.01) years |
|  |  |  |
| **Brain functional integrity measures** | | |
|  | **Objective Cognitive Function:**  Full-scale IQ | Objective cognitive function was assessed using the Wechsler Adult Intelligence Scale–IV (WAIS-IV; potential score range 40-160; standardized in the full cohort to mean[SD]=100[15]) at age 45 years to produce the overall full-scale IQ. Higher scores indicate greater cognitive performance.  Analytic sample, N = 918, M mean(SD) = 100 (15). |
|  | **Subjective Cognitive Function:**  Everyday cognitive problems | Subjective everyday cognitive function was reported on by the Study members (self-report) and by individuals who knew the Study members well (informant-report). With matched checklist items, the Study members were interviewed by trained Study staff and the informants were mailed questionnaires. Both were asked about whether the Study members had problems with their memory or attention over the past year, including questions such as whether they were more likely to be “easily distracted” and “get sidetracked” as well as to “misplace wallet, keys or eyeglasses” and “forget to do errands, return calls or pay bills.” Self and informant reports were correlated at r=.38, *p*<.001. Self and informant memory and attention scales were z-scored and averaged to produce an overall subject scale of problems with everyday cognitive function.  Analytic sample, N = 921, M (SD) = 0.01 (0.77). |
|  | **Cognitive Decline:**  Residualized cognitive change from childhood | Cognitive change (decline) was assessed as change in objective cognitive function from childhood to midlife measured by studying IQ scores at midlife after controlling for IQ scores in childhood (“residualized change”). The Wechsler Intelligence Scale for Children–Revised (WISC–R; potential score range 40-160; standardized in the full cohort to mean(SD)=100(15)]) yielding the full-scale IQ was administered to each participant at ages 7, 9, and 11 years, with scores from all years averaged together to increase baseline reliability.  Analytic sample, N = 906, M (SD) = 0.00 (9.55). |

*Note.* SD = standard deviation.

# **Appendix 3.** Ascertainment of individual-level socioeconomic status in the Dunedin Study.

Individual-level socioeconomic status was derived from information about each Study member’s occupational status at age 45 years. Occupations were scored using the New Zealand Socioeconomic Index (NZSEI-06), which codes each occupation based on its associated education level and income in the NZ Census (score range, 10 [low status]-90 [high status]) and groups occupations into 6 status levels. Examples of occupations in the 6 groups include medical practitioner (NZSEI code 90; group 6), engineering professional (code 66; group 5), database administrator (code 59; group 4), personal assistant (code 44; group 3), office cashier (code 28; group 2), and fish filleter (code 23; group 1). Homemakers and others not working in the past year were assigned the status of their most recent occupation, as reported at age 38. Study members who had been out of the labor force since age 32 were assigned the status of their partner if they had a partner with whom they shared a household; if they did not share a household with a partner, their socioeconomic status was assigned based on their education level.

# **Table S6.** Prevalence of dementia in the NZ-IDI.

|  | **Dementia prevalence** | | | | | | | |
| --- | --- | --- | --- | --- | --- | --- | --- | --- |
|  | **Overall** | |  | **Male** | |  | **Female** | |
|  | **N** | **%** |  | **N** | **%** |  | **N** | **%** |
| **Total** (N=1,695,447) | 36,753 | 2.17 |  | 18,213 | 2.13 |  | 18,540 | 2.20 |
|  |  |  |  |  |  |  |  |  |
| **Born 1929-38** (N=231,567) | 24,279 | 10.48 |  | 11,391 | 10.04 |  | 12,888 | 10.91 |
| **Born 1939-48** (N=361,200) | 9,414 | 2.61 |  | 5,073 | 2.79 |  | 4,341 | 2.42 |
| **Born 1949-58** (N=504,372) | 2,436 | 0.48 |  | 1,392 | 0.54 |  | 1,044 | 0.42 |
| **Born 1959-68** (N=598,308) | 624 | 0.10 |  | 357 | 0.12 |  | 267 | 0.09 |

*Note*. Counts were randomly rounded to a base of three per the confidentiality rules of Statistics New Zealand. Although dementia in the community was likely under-identified in our medical register–based ascertainment scheme, cases were classified accurately: 83.1%of cases diagnosed with dementia in medical registers were also diagnosed in community-based assessments (eAppendix 3 in the Supplement of Richmond-Rakerd et al., 2022).^32^

# **Table S7.** Distribution of neighborhood disadvantage at first residential address in the NZ-IDI.

|  | **Mean neighborhood disadvantage quintile score, by age cohort and sex** | |  | **N at each quintile of neighborhood disadvantage, by age cohort**  **(row %)** | | | | |
| --- | --- | --- | --- | --- | --- | --- | --- | --- |
|  | |  |  | Q1 | Q2 | Q3 | Q4 | Q5 |
| Total population | 2.97 | |  | 285,516 | 285,432 | 286,629 | 288,021 | 263,223 |
|  |  |  |  | (20.3%) | (20.3%) | (20.3%) | (20.4%) | (18.7%) |
|  |  |  |  |  |  |  |  |  |
|  | **Male** | **Female** |  |  |  |  |  |  |
| Born 1929-38 | 3.05 | 3.11 |  | 32,082 | 36,003 | 39,555 | 43,104 | 36,033 |
|  |  |  |  | (17.2%) | (19.3%) | (21.2%) | (23.1%) | (19.3%) |
| Born 1939-48 | 2.91 | 2.95 |  | 60,024 | 59,658 | 58,764 | 58,290 | 50,694 |
|  |  |  |  | (20.9%) | (20.8%) | (20.4%) | (20.3%) | (17.6%) |
| Born 1949-58 | 2.91 | 2.93 |  | 87,987 | 84,585 | 82,458 | 80,958 | 73,722 |
|  |  |  |  | (21.5%) | (20.6%) | (20.1%) | (19.8%) | (18.0%) |
| Born 1959-68 | 2.99 | 2.99 |  | 105,423 | 105,186 | 105,852 | 105,669 | 102,774 |
|  |  |  |  | (20.1%) | (20.0%) | (20.2%) | (20.1%) | (19.6%) |
|  |  |  |  |  |  |  |  |  |

*Note.* Q1 = low disadvantage, Q5 = high disadvantage. Counts were randomly rounded to a base of three per the confidentiality rules of Statistics New Zealand.

# **Appendix 4**. Age-45 Data Collection Wave attrition analysis.

We conducted an attrition analysis using childhood IQ, childhood SES, a lifelong history of mental health problems from ages 18-45 years (the p-factor),^107^ Adverse Childhood Experiences (ACEs), and a polygenic score for educational attainment to determine whether participants in the Phase 45 data collection were representative of the original cohort.

No significant differences in childhood IQ were found between the full cohort, those still alive, those seen at Phase 45 or those who underwent brain scans at Phase 45. Those who were deceased by the Phase 45 data collection had significantly lower childhood IQ’s than those who were still alive (t = 2.09, p = 0.04).


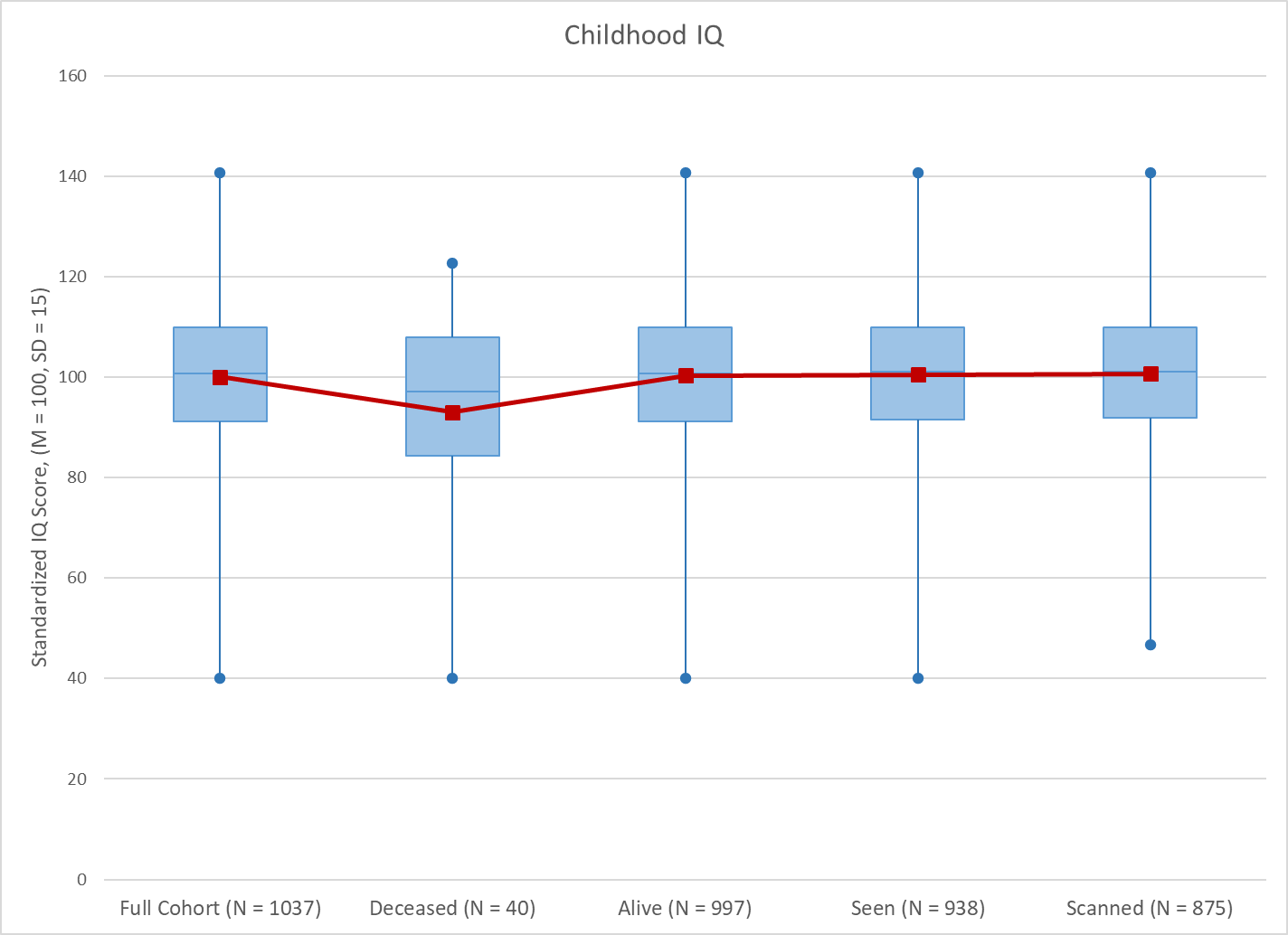


No significant differences were found between the full cohort, those deceased, those alive, those seen at Phase 45 or those scanned at Phase 45 on childhood SES.


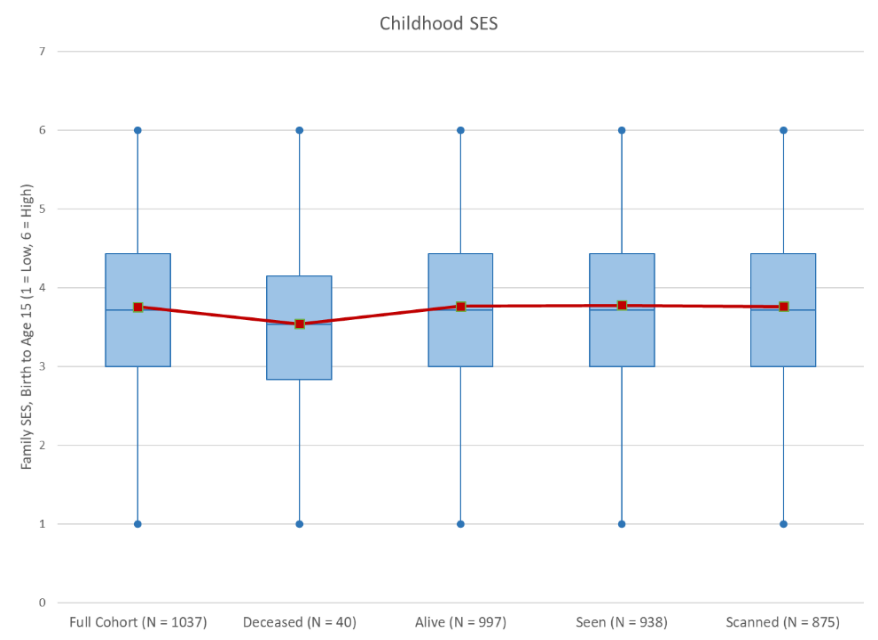


No significant differences in p-factor were found between the full cohort, those still alive, those seen at Phase 45 or those scanned at Phase 45. Those who were deceased by the Phase 45 data collection had significantly higher p-factor scores than those who were still alive (t = -2.86, p = 0.004).


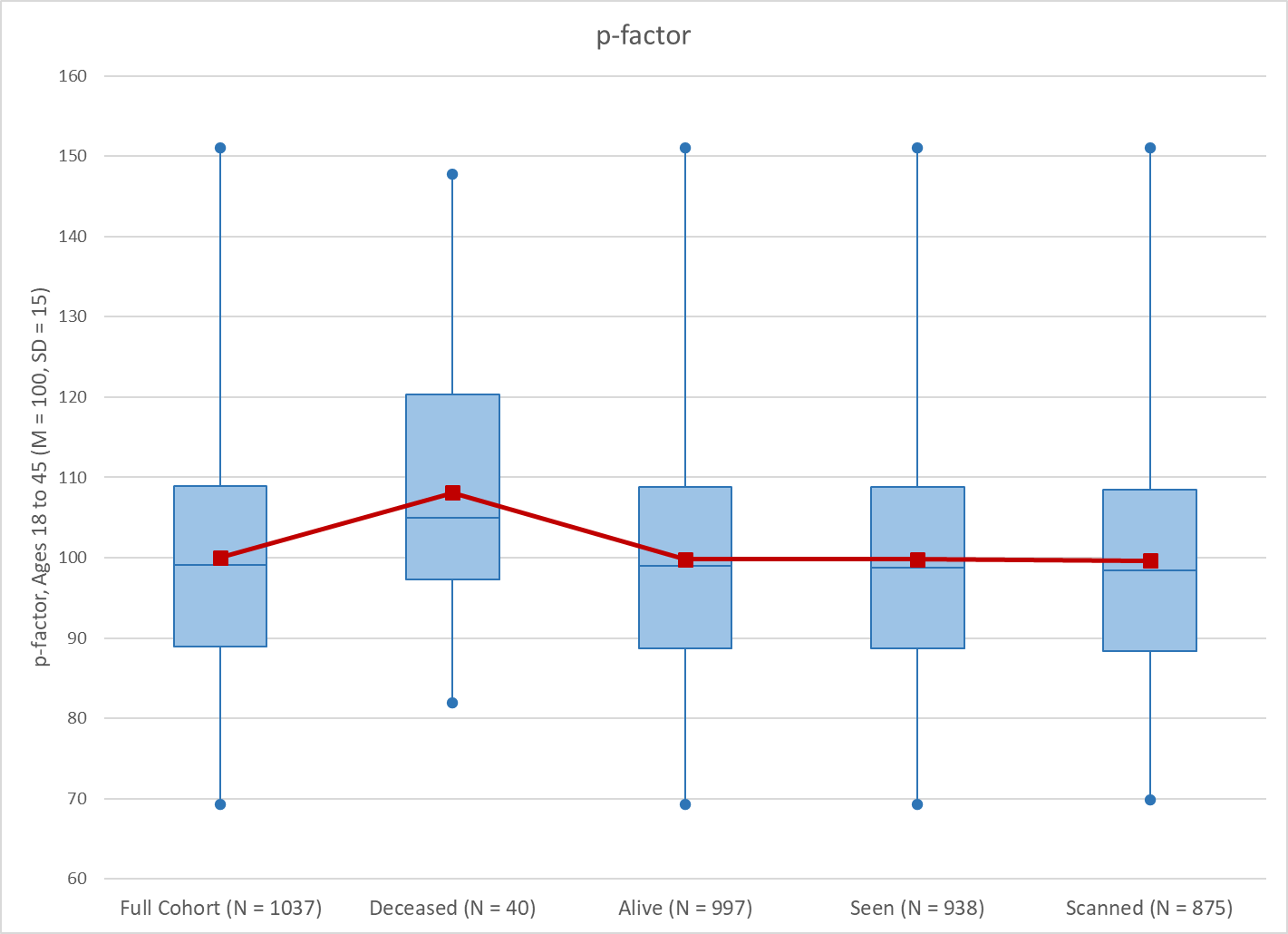


No significant differences were found between the full cohort, those deceased, those alive, those seen at Phase 45 or those scanned at Phase 45 on Adverse Childhood Experiences (ACEs).


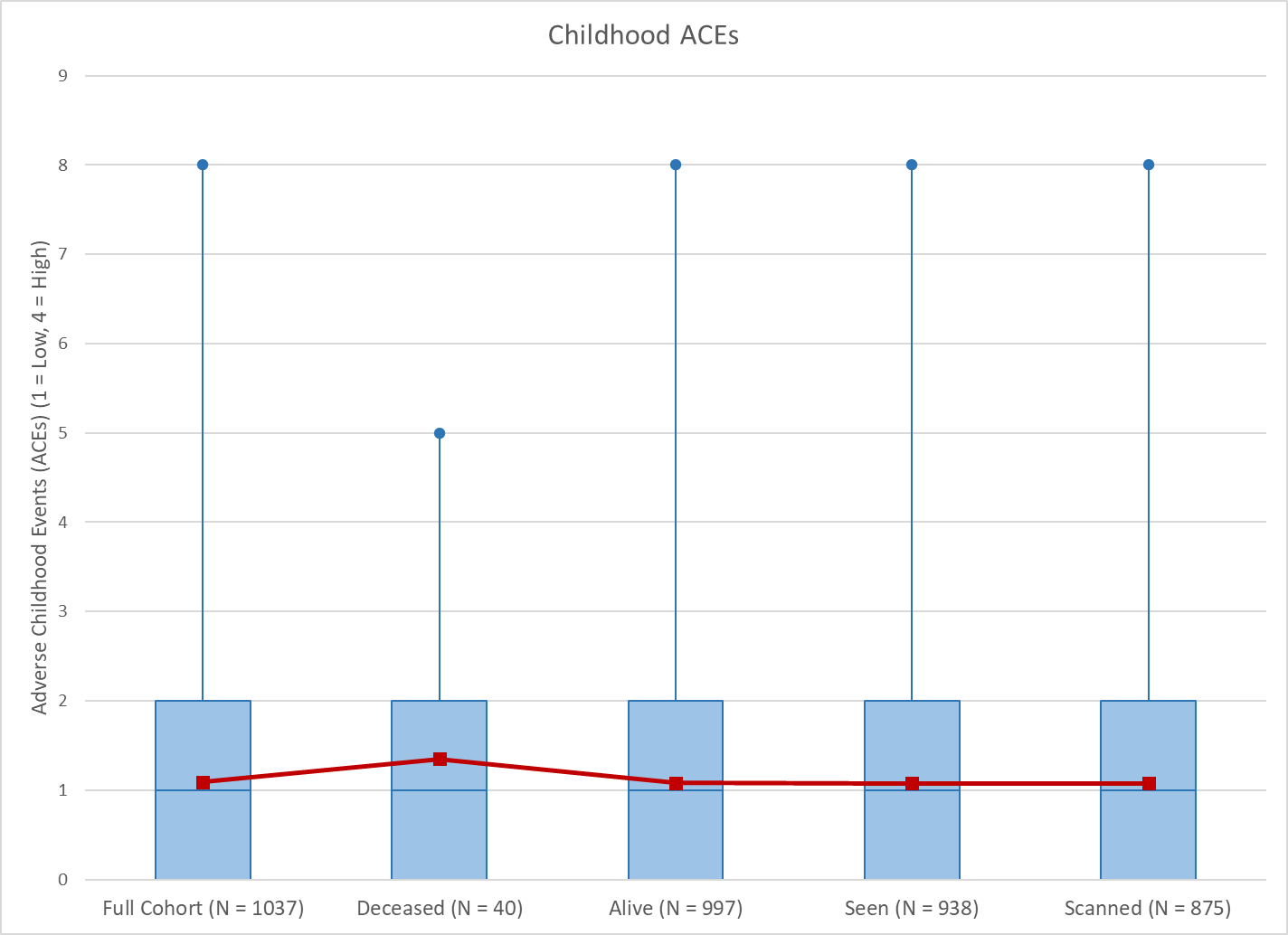


No significant differences were found between the full cohort, those deceased, those alive, those seen at Phase 45 or those scanned at Phase 45 on Childhood Low Self-Control.


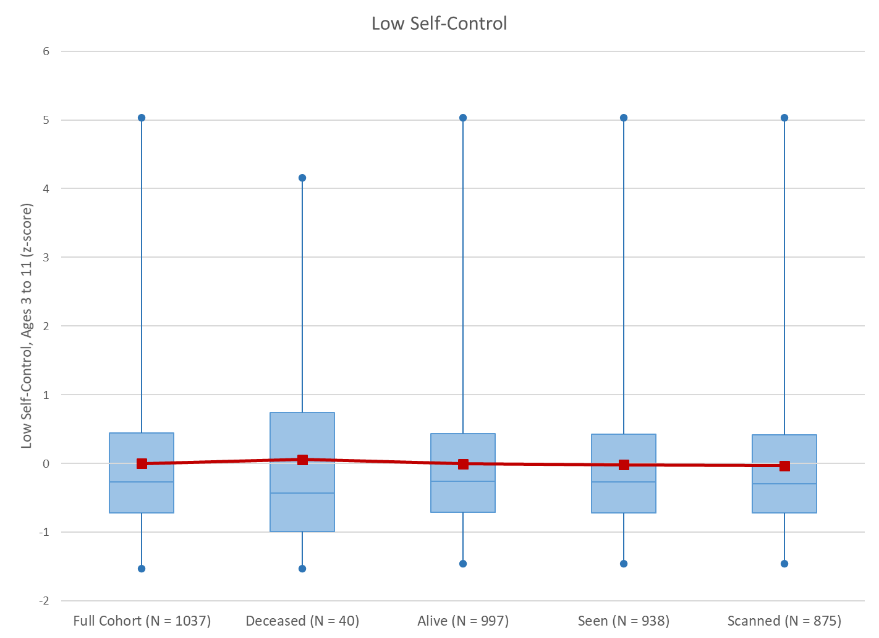


We began collecting DNA from Study members at age 26, in 1998. The DNA biobank does not contain DNA from Study members of Maori descent. No significant differences were found between non-Maori participants with DNA, those who subsequently died, those alive, those seen at Phase 45 or those scanned at Phase 45 on the SSGAC 2021 polygenic score for educational attainment.


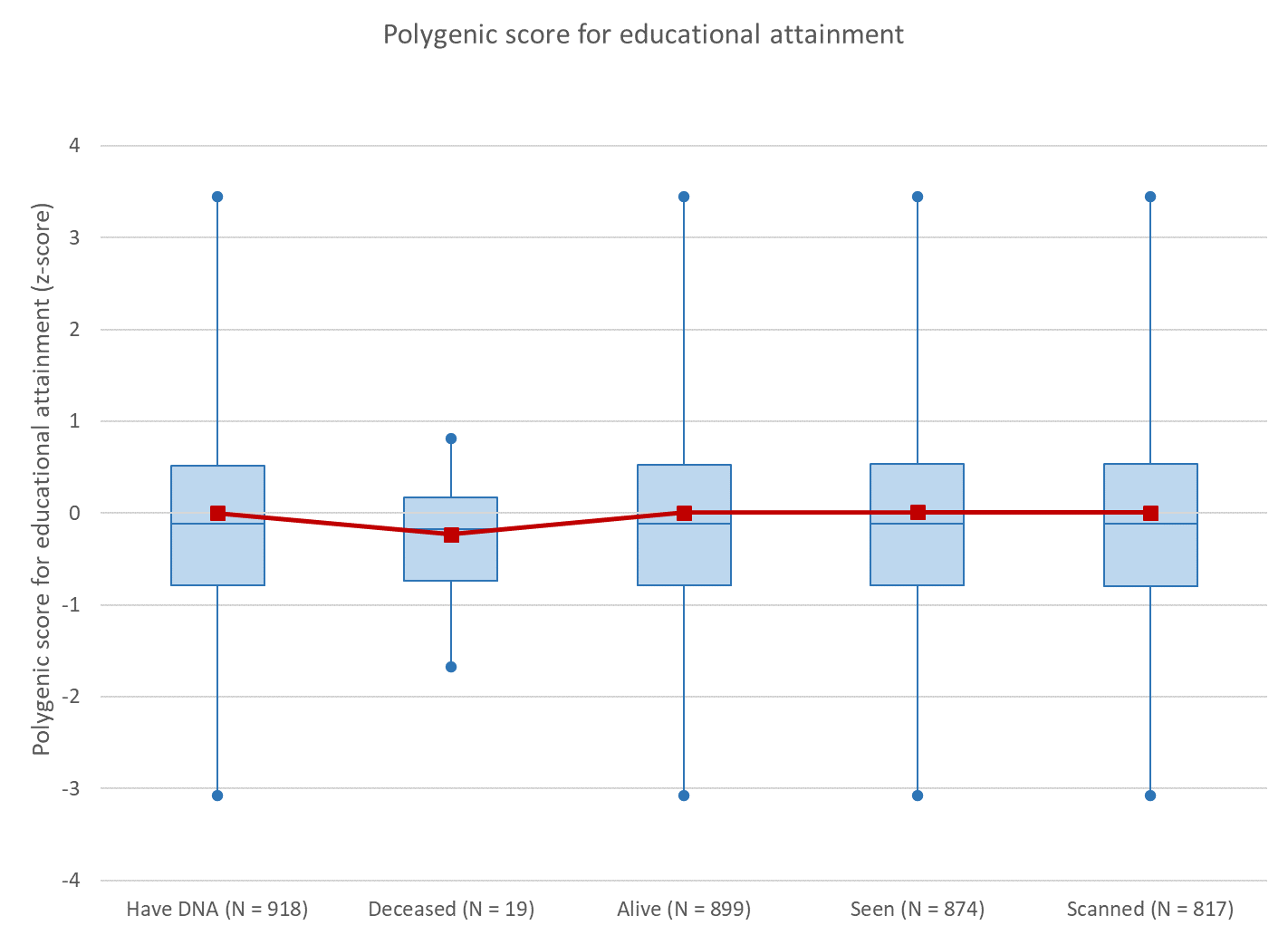


# **Figure S2.** The distribution of neighborhood disadvantage in the Dunedin Cohort, from ages 26 to 45.

**Panel A.** The distribution of cumulative adult neighborhood disadvantage (ages 26 to 45 years) (in z-score units).

**Panel B.** The distribution of raw neighborhood deprivation (NZDep) decile scores at each assessment wave, from ages 26 to 45 years.

*Note.* 1= least deprived decile, 10 = most deprived.

# **Table S8.** Association of neighborhood disadvantage with the 10 domains of risk comprising the Dunedin ADRD Risk Benchmark

|  | **Association with neighborhood disadvantage (sex adjusted)** | |  | **Association with neighborhood disadvantage (fully adjusted)** | |
| --- | --- | --- | --- | --- | --- |
|  | **β** | ***p*-value** |  | **β** | ***p*-value** |
| **1. Genetic risk** | .02 | .504 |  | .03 | .415 |
| **2. Lifestyle risk** | .22 | <.001 |  | .13 | <.001 |
| **3. Social class risk** | .33 | <.001 |  | .06 | .001 |
| **4. Psycho-somatic risk** | .20 | <.001 |  | .13 | <.001 |
| **5. Physical & sensory risk** | .22 | <.001 |  | .15 | <.001 |
| **6. Cardio-metabolic risk** | .12 | .005 |  | .10 | .004 |
| **7. Inflammatory risk** | .18 | <.001 |  | .15 | <.001 |
| **8. DNA methylation aging risk** | .16 | <.001 |  | .12 | .<.001 |
| **9. Harmful events risk** | .08 | .009 |  | .05 | .139 |
| **10. Subjective health risk** | .35 | <.001 |  | .25 | <.001 |

*Note*: Fully adjusted associations include sex and the covariate of individual-level socioeconomic status.

# **References**

1. Aneshensel CS, Ko MJ, Chodosh J, Wight RG. The urban neighborhood and cognitive functioning in late middle age. *J Health Soc Behav*. 2011;52(2):163-179. doi:10.1177/0022146510393974

2. Becerril A, Pfoh ER, Hashmi AZ, et al. Racial, ethnic and neighborhood socioeconomic differences in incidence of dementia: A regional retrospective cohort study. *J Am Geriatr Soc*. Published online 2023:1-13. doi:10.1111/jgs.18322

3. Cadar D, Lassale C, Davies H, Llewellyn DJ, Batty GD, Steptoe A. Individual and Area-Based Socioeconomic Factors Associated With Dementia Incidence in England: Evidence From a 12-Year Follow-up in the English Longitudinal Study of Ageing. *JAMA Psychiatry*. 2018;75(7):723-732. doi:10.1001/jamapsychiatry.2018.1012

4. Chamberlain AM, St. Sauver JL, Finney Rutten LJ, et al. Associations of neighborhood socioeconomic disadvantage With chronic conditions by age, sex, race, and ethnicity in a population-based cohort. *Mayo Clin Proc*. 2022;97(1):57-67. doi:10.1016/j.mayocp.2021.09.006

5. Clarke PJ, Weuve J, Barnes L, Evans DA, Mendes de Leon CF. Cognitive decline and the neighborhood environment. *Ann Epidemiol*. 2015;25(11):849-854. doi:10.1016/j.annepidem.2015.07.001

6. Dintica CS, Bahorik A, Xia F, Kind A, Yaffe K. Dementia Risk and disadvantaged neighborhoods. *JAMA Neurol*. Published online July 19, 2023. doi:10.1001/jamaneurol.2023.2120

7. George KM, Lutsey PL, Kucharska-Newton A, et al. Life-Course Individual and Neighborhood Socioeconomic Status and Risk of Dementia in the Atherosclerosis Risk in Communities Neurocognitive Study. *Am J Epidemiol*. 2020;189(10):1134-1142. doi:10.1093/aje/kwaa072

8. Hamilton CA, Matthews FE, Erskine D, Attems J, Thomas AJ. Neurodegenerative brain changes are associated with area deprivation in the United Kingdom: findings from the Brains for Dementia Research study. *Acta Neuropathol Commun*. 2021;9(1):198. doi:10.1186/s40478-021-01301-8

9. Hunt JFV, Buckingham W, Kim AJ, et al. Association of neighborhood-level disadvantage with cerebral and hippocampal volume. *JAMA Neurol*. 2020;77(4):451-460. doi:10.1001/jamaneurol.2019.4501

10. Hunt JFV, Vogt NM, Jonaitis EM, et al. Association of neighborhood context, cognitive decline, and cortical change in an unimpaired cohort. *Neurology*. 2021;96(20):e2500-e2512. doi:10.1212/WNL.0000000000011918

11. Kim MH, Foverskov E, Frøslev T, et al. Neighborhood disadvantage and the risk of dementia and mortality among refugees to Denmark: A quasi-experimental study. *SSM - Popul Health*. 2023;21:101312. doi:10.1016/j.ssmph.2022.101312

12. Kuchibhatla M, Hunter JC, Plassman BL, et al. The association between neighborhood socioeconomic status, cardiovascular and cerebrovascular risk factors, and cognitive decline in the Health and Retirement Study (HRS). *Aging Ment Health*. 2020;24(9):1479-1486. doi:10.1080/13607863.2019.1594169

13. Marsh K, Shao Y, Zhang Y, Masurkar AV, Vedvyas A, Chodosh J. Association of neighborhood socioeconomic disadvantage and cognitive decline. *Alzheimers Dement*. 2021;17(S10):e056584. doi:10.1002/alz.056584

14. McCann A, McNulty H, Rigby J, et al. Effect of Area-Level Socioeconomic Deprivation on Risk of Cognitive Dysfunction in Older Adults. *J Am Geriatr Soc*. 2018;66(7):1269-1275. doi:10.1111/jgs.15258

15. Merkel EX, Ennis GE, Cadman RV, et al. Testing the Association Between Neighborhood Disadvantage and White Matter Hyperintensities. *Alzheimers Dement*. 2022;18(S11):e069324. doi:10.1002/alz.069324

16. Meyer OL, Mungas D, King J, et al. Neighborhood Socioeconomic Status and Cognitive Trajectories in a Diverse Longitudinal Cohort. *Clin Gerontol*. 2018;41(1):82-93. doi:10.1080/07317115.2017.1282911

17. Meyer OL, Besser L, Tobias M, et al. Neighborhood socioeconomic status and segregation linked to cognitive decline. *Alzheimers Dement Diagn Assess Dis Monit*. 2023;15(1):e12401. doi:10.1002/dad2.12401

18. Mobley TM, Shaw C, Hayes-Larson E, et al. Neighborhood disadvantage and dementia incidence in a cohort of Asian American and non-Latino White older adults in Northern California. *Alzheimers Dement J Alzheimers Assoc*. 2023;19(1):296-306. doi:10.1002/alz.12660

19. Ouvrard C, Meillon C, Dartigues JF, Ávila-Funes JA, Amieva H. Do Individual and Geographical Deprivation Have the Same Impact on the Risk of Dementia? A 25-Year Follow-up Study. *J Gerontol Ser B*. 2020;75(1):218-227. doi:10.1093/geronb/gbx130

20. Pase MP, Rowsthorn E, Cavuoto MG, et al. Association of Neighborhood-Level Socioeconomic Measures With Cognition and Dementia Risk in Australian Adults. *JAMA Netw Open*. 2022;5(3):e224071. doi:10.1001/jamanetworkopen.2022.4071

21. Powell WR, Buckingham WR, Larson JL, et al. Association of neighborhood-level disadvantage with Alzheimer disease neuropathology. *JAMA Netw Open*. 2020;3(6):e207559. doi:10.1001/jamanetworkopen.2020.7559

22. Powell WR, Zuelsdorff M, Keller SA, et al. Association of Neighborhood-Level Disadvantage With Neurofibrillary Tangles on Neuropathological Tissue Assessment. *JAMA Netw Open*. 2022;5(4):e228966. doi:10.1001/jamanetworkopen.2022.8966

23. Rosso AL, Flatt JD, Carlson MC, et al. Neighborhood Socioeconomic Status and Cognitive Function in Late Life. *Am J Epidemiol*. 2016;183(12):1088-1097. doi:10.1093/aje/kwv337

24. Sheffield KM, Peek MK. Neighborhood context and cognitive decline in older Mexican Americans: results from the Hispanic Established Populations for Epidemiologic Studies of the Elderly. *Am J Epidemiol*. 2009;169(9):1092-1101. doi:10.1093/aje/kwp005

25. Shih RA, Ghosh-Dastidar B, Margolis KL, et al. Neighborhood socioeconomic status and cognitive function in women. *Am J Public Health*. 2011;101(9):1721-1728. doi:10.2105/AJPH.2011.300169

26. Tan CH, Tan JJX. Low neighborhood deprivation buffers against hippocampal neurodegeneration, white matter hyperintensities, and poorer cognition. *GeroScience*. Published online April 1, 2023. doi:10.1007/s11357-023-00780-y

27. Thierry AD, Sherman-Wilkins K, Armendariz M, Sullivan A, Farmer HR. Perceived neighborhood characteristics and cognitive functioning among diverse older adults: An intersectional approach. *Int J Environ Res Public Health*. 2021;18(5). doi:10.3390/ijerph18052661

28. Vassilaki M, Aakre JA, Castillo A, et al. Association of neighborhood socioeconomic disadvantage and cognitive impairment. *Alzheimers Dement J Alzheimers Assoc*. Published online June 6, 2022. doi:10.1002/alz.12702

29. Wight RG, Aneshensel CS, Miller-Martinez D, et al. Urban neighborhood context, educational attainment, and cognitive function among older adults. *Am J Epidemiol*. 2006;163(12):1071-1078. doi:10.1093/aje/kwj176

30. Stats NZ Geographic Data Service. Statistical Area 1 2021 (generalised). Accessed April 12, 2023. https://datafinder.stats.govt.nz/layer/105162-statistical-area-1-2021-generalised/

31. Australian Bureau of Statistics. The Index of Relative Socio-economic Advantage and Disadvantage. Published 2018. Accessed April 13, 2023. https://www.abs.gov.au/ausstats/abs@.nsf/Lookup/by%20Subject/2033.0.55.001~2016~Main%20Features~IRSAD~20

32. Richmond-Rakerd LS, D’Souza S, Milne BJ, Caspi A, Moffitt TE. Longitudinal associations of mental disorders with dementia: 30-year analysis of 1.7 million New Zealand citizens. *JAMA Psychiatry*. 2022;79(4):333-340. doi:10.1001/jamapsychiatry.2021.4377

33. Walesby KE, Exeter DJ, Gibb S, Wood PC, Starr JM, Russ TC. Prevalence and geographical variation of dementia in New Zealand from 2012 to 2015: Brief report utilising routinely collected data within the Integrated Data Infrastructure. *Australas J Ageing*. 2020;39(3):297-304. doi:10.1111/ajag.12790

34. Kivipelto M, Ngandu T, Laatikainen T, Winblad B, Soininen H, Tuomilehto J. Risk score for the prediction of dementia risk in 20 years among middle aged people: a longitudinal, population-based study. *Lancet Neurol*. 2006;5(9):735-741. doi:10.1016/S1474-4422(06)70537-3

35. Vos SJB, van Boxtel MPJ, Schiepers OJG, et al. Modifiable risk Factors for prevention of dementia in midlife, late life and the oldest-old: Validation of the LIBRA index. *J Alzheimers Dis*. 2017;58(2):537-547. doi:10.3233/JAD-161208

36. Livingston G, Huntley J, Sommerlad A, et al. Dementia prevention, intervention, and care: 2020 report of the Lancet Commission. *The Lancet*. 2020;396(10248):413-446. doi:10.1016/S0140-6736(20)30367-6

37. Anstey KJ, Cherbuin N, Herath PM. Development of a new method for assessing global risk of Alzheimer’s disease for use in population health approaches to prevention. *Prev Sci*. 2013;14(4):411-421. doi:10.1007/s11121-012-0313-2

38. Russell D, Peplau LA, Cutrona CE. The revised UCLA Loneliness Scale: Concurrent and discriminant validity evidence. *J Pers Soc Psychol*. 1980;39(3):472-480. doi:10.1037/0022-3514.39.3.472

39. Huang W, Qiu C, von Strauss E, Winblad B, Fratiglioni L. APOE genotype, family history of dementia, and Alzheimer disease risk: A 6-year follow-up study. *Arch Neurol*. 2004;61(12):1930-1934. doi:10.1001/archneur.61.12.1930

40. Rawle MJ, Davis D, Bendayan R, Wong A, Kuh D, Richards M. Apolipoprotein-E (Apoe) ε4 and cognitive decline over the adult life course. *Transl Psychiatry*. 2018;8(1):1-8. doi:10.1038/s41398-017-0064-8

41. Freedman VA, Kasper JD. Cohort Profile: The National Health and Aging Trends Study (NHATS). *Int J Epidemiol*. 2019;48(4):1044-1045g. doi:10.1093/ije/dyz109

42. ALzGene. Meta-analysis of all published AD association studies (case-control only) APOE E2/3/4. Published 2010. Accessed May 25, 2022. http://www.alzgene.org/meta.asp?geneID=83

43. Scarmeas N, Luchsinger JA, Schupf N, et al. Physical activity, diet, and risk of Alzheimer disease. *JAMA J Am Med Assoc*. 2009;302(6):627-637. doi:10.1001/jama.2009.1144

44. Tyndall AV, Clark CM, Anderson TJ, et al. Protective effects of exercise on cognition and brain health in older adults. *Exerc Sport Sci Rev*. 2018;46(4):215-223. doi:10.1249/JES.0000000000000161

45. Durazzo TC, Mattsson N, Weiner MW. Smoking and increased Alzheimer’s disease risk: A review of potential mechanisms. *Alzheimers Dement J Alzheimers Assoc*. 2014;10(3 0):S122-S145. doi:10.1016/j.jalz.2014.04.009

46. Ballarini T, Schröder A, Lent DM van, et al. The effects of Mediterranean diet on memory and Alzheimer’s disease biomarkers. *Alzheimers Dement*. 2020;16(S10):e045349. doi:https://doi.org/10.1002/alz.045349

47. Lefèvre-Arbogast S, Féart C, Dartigues JF, Helmer C, Letenneur L, Samieri C. Dietary B vitamins and a 10-year risk of dementia in older persons. *Nutrients*. 2016;8(12):761. doi:10.3390/nu8120761

48. Szekely CA, Breitner JCS, Fitzpatrick AL, et al. NSAID use and dementia risk in the Cardiovascular Health Study: Role of APOE and NSAID type. *Neurology*. 2008;70(1):17-24. doi:10.1212/01.wnl.0000284596.95156.48

49. World Health Organization. *WHO Guidelines on Physical Activity and Sedentary Behaviour*.; 2020.

50. Anstey KJ, Cherbuin N, Herath PM, et al. A self-report risk index to predict occurrence of dementia in three independent cohorts of older adults: The ANU-ADRI. *PLOS ONE*. 2014;9(1):e86141. doi:10.1371/journal.pone.0086141

51. Schiepers OJG, Köhler S, Deckers K, et al. Lifestyle for Brain Health (LIBRA): a new model for dementia prevention. *Int J Geriatr Psychiatry*. 2018;33(1):167-175. doi:10.1002/gps.4700

52. Seblova D, Fischer M, Fors S, et al. Does prolonged education causally affect dementia risk when adult socioeconomic status is not altered? A Swedish natural experiment in 1.3 million individuals. *Am J Epidemiol*. 2021;190(5):817-826. doi:10.1093/aje/kwaa255

53. Russ TC, Stamatakis E, Hamer M, Starr JM, Kivimäki M, Batty GD. Socioeconomic status as a risk factor for dementia death: individual participant meta-analysis of 86 508 men and women from the UK. *Br J Psychiatry*. 2013;203(1):10-17. doi:10.1192/bjp.bp.112.119479

54. Sharp ES, Gatz M. The relationship between education and dementia an updated systematic review. *Alzheimer Dis Assoc Disord*. 2011;25(4):289-304. doi:10.1097/WAD.0b013e318211c83c

55. Richmond-Rakerd LS, D’Souza S, Andersen SH, et al. Clustering of health, crime and social-welfare inequality in 4 million citizens from two nations. *Nat Hum Behav*. Published online January 20, 2020:1-10. doi:10.1038/s41562-019-0810-4

56. Low LF, Harrison F, Lackersteen SM. Does personality affect risk for dementia? A systematic review and meta-analysis. *Am J Geriatr Psychiatry*. 2013;21(8):713-728. doi:10.1016/j.jagp.2012.08.004

57. Sabia S, Fayosse A, Dumurgier J, et al. Association of sleep duration in middle and old age with incidence of dementia. *Nat Commun*. 2021;12(1):2289. doi:10.1038/s41467-021-22354-2

58. Zilkens RR, Bruce DG, Duke J, Spilsbury K, Semmens JB. Severe psychiatric disorders in mid-Life and risk of dementia in late-life (Age 65-84 Years): A population based case-control Study. *Curr Alzheimer Res*. 2014;11(7):681-693. doi:10.2174/1567205011666140812115004

59. Spira AP, Chen-Edinboro LP, Wu MN, Yaffe K. Impact of sleep on the risk of cognitive decline and dementia. *Curr Opin Psychiatry*. 2014;27(6):478-483. doi:10.1097/YCO.0000000000000106

60. Chuang CS, Lin CL, Lin MC, Sung FC, Kao CH. Migraine and risk of dementia: A nationwide retrospective cohort study. *Neuroepidemiology*. 2013;41(3-4):139-145. doi:10.1159/000353559

61. Islamoska S, Hansen ÅM, Wang HX, et al. Mid- to late-life migraine diagnoses and risk of dementia: a national register-based follow-up study. *J Headache Pain*. 2020;21(1):98. doi:10.1186/s10194-020-01166-7

62. Headache Classification Committee of the International Headache Society. Classification and diagnostic criteria for headache disorders, cranial neuralgias and facial pain. *Cephalalgia*. 1988;8 Suppl 7:1-96.

63. Waldie KE, Hausmann M, Milne BJ, Poulton R. Migraine and cognitive function: a life-course study. *Neurology*. 2002;59(6):904-908. doi:10.1212/wnl.59.6.904

64. Buysse DJ, Reynolds CF, Monk TH, Berman SR, Kupfer DJ. The Pittsburgh Sleep Quality Index: a new instrument for psychiatric practice and research. *Psychiatry Res*. 1989;28(2):193-213. doi:10.1016/0165-1781(89)90047-4

65. Benet-Martínez V, John OP. Los Cinco Grandes across cultures and ethnic groups: multitrait multimethod analyses of the Big Five in Spanish and English. *J Pers Soc Psychol*. 1998;75(3):729-750.

66. Verghese J, Lipton RB, Hall CB, Kuslansky G, Katz MJ, Buschke H. Abnormality of gait as a predictor of non-Alzheimer’s dementia. *N Engl J Med*. 2002;347(22):1761-1768. doi:10.1056/NEJMoa020441

67. Thomson RS, Auduong P, Miller AT, Gurgel RK. Hearing loss as a risk factor for dementia: A systematic review. *Laryngoscope Investig Otolaryngol*. 2017;2(2):69-79. doi:10.1002/lio2.65

68. Paik JS, Ha M, Jung YH, et al. Low vision and the risk of dementia: a nationwide population-based cohort study. *Sci Rep*. 2020;10(1):9109. doi:10.1038/s41598-020-66002-z

69. Bathini P, Brai E, Auber LA. Olfactory dysfunction in the pathophysiological continuum of dementia. *Ageing Res Rev*. 2019;55:100956. doi:10.1016/j.arr.2019.100956

70. Verghese J, Wang C, Lipton RB, Holtzer R, Xue X. Quantitative gait dysfunction and risk of cognitive decline and dementia. *J Neurol Neurosurg Psychiatry*. 2007;78(9):929-935. doi:10.1136/jnnp.2006.106914

71. Naël V, Pérès K, Dartigues JF, et al. Vision loss and 12-year risk of dementia in older adults: the 3C cohort study. *Eur J Epidemiol*. 2019;34(2):141-152. doi:10.1007/s10654-018-00478-y

72. Springer BA, Marin R, Cyhan T, Roberts H, Gill NW. Normative values for the unipedal stance test with eyes open and closed. *J Geriatr Phys Ther 2001*. 2007;30(1):8-15. doi:10.1519/00139143-200704000-00003

73. Rasmussen LJH, Caspi A, Ambler A, et al. Association of neurocognitive and physical function with gait speed in midlife. *JAMA Netw Open*. 2019;2(10):e1913123. doi:10.1001/jamanetworkopen.2019.13123

74. Noble W, Jensen NS, Naylor G, Bhullar N, Akeroyd MA. A short form of the Speech, Spatial and Qualities of Hearing scale suitable for clinical use: the SSQ12. *Int J Audiol*. 2013;52(6):409-412. doi:10.3109/14992027.2013.781278

75. Frost NA, Sparrow JM, Durant JS, Donovan JL, Peters TJ, Brookes ST. Development of a questionnaire for measurement of vision-related quality of life. *Ophthalmic Epidemiol*. 1998;5(4):185-210. doi:10.1076/opep.5.4.185.4191

76. Exalto LG, Whitmer RA, Kappele LJ, Biessels GJ. An update on type 2 diabetes, vascular dementia and Alzheimer’s disease. *Exp Gerontol*. 2012;47(11):858-864. doi:10.1016/j.exger.2012.07.014

77. Ninomiya T. Epidemiological evidence of the relationship between diabetes and dementia. In: Nakabeppu Y, Ninomiya T, eds. *Diabetes Mellitus: A Risk Factor for Alzheimer’s Disease*. Advances in Experimental Medicine and Biology. Springer; 2019:13-25. doi:10.1007/978-981-13-3540-2_2

78. Perrotta M, Lembo G, Carnevale D. Hypertension and dementia: epidemiological and experimental evidence revealing a detrimental relationship. *Int J Mol Sci*. 2016;17(3):347. doi:10.3390/ijms17030347

79. Wartolowska KA, Webb AJS. Midlife blood pressure is associated with the severity of white matter hyperintensities: analysis of the UK Biobank cohort study. *Eur Heart J*. 2021;42(7):750-757. doi:10.1093/eurheartj/ehaa756

80. Cheung CY, Chan VTT, Mok VC, Chen C, Wong TY. Potential retinal biomarkers for dementia: what is new? *Curr Opin Neurol*. 2019;32(1):82-91. doi:10.1097/WCO.0000000000000645

81. Jong FJ de, Schrijvers EMC, Ikram MK, et al. Retinal vascular caliber and risk of dementia: The Rotterdam Study. *Neurology*. 2011;76(9):816-821. doi:10.1212/WNL.0b013e31820e7baa

82. Cheung CY, Tay WT, Mitchell P, et al. Quantitative and qualitative retinal microvascular characteristics and blood pressure. *J Hypertens*. 2011;29(7):1380-1391. doi:10.1097/HJH.0b013e328347266c

83. Peila R, Launer LJ. Inflammation and dementia: epidemiologic evidence. *Acta Neurol Scand Suppl*. 2006;185:102-106. doi:10.1111/j.1600-0404.2006.00693.x

84. Schmidt R, Schmidt H, Curb JD, Masaki K, White LR, Launer LJ. Early inflammation and dementia: a 25-year follow-up of the Honolulu-Asia Aging Study. *Ann Neurol*. 2002;52(2):168-174. doi:10.1002/ana.10265

85. Kinney JW, Bemiller SM, Murtishaw AS, Leisgang AM, Salazar AM, Lamb BT. Inflammation as a central mechanism in Alzheimer’s disease. *Alzheimers Dement N Y N*. 2018;4:575-590. doi:10.1016/j.trci.2018.06.014

86. Salameh Y, Bejaoui Y, El Hajj N. DNA methylation biomarkers in aging and age-related diseases. *Front Genet*. 2020;11:171. doi:10.3389/fgene.2020.00171

87. Fransquet PD, Lacaze P, Saffery R, McNeil J, Woods R, Ryan J. Blood DNA methylation as a potential biomarker of dementia: A systematic review. *Alzheimers Dement*. 2018;14(1):81-103. doi:10.1016/j.jalz.2017.10.002

88. Horvath S. DNA methylation age of human tissues and cell types. *Genome Biol*. 2013;14(10):3156. doi:10.1186/gb-2013-14-10-r115

89. Hannum G, Guinney J, Zhao L, et al. Genome-wide methylation profiles reveal quantitative views of human aging rates. *Mol Cell*. 2013;49(2):359-367. doi:10.1016/j.molcel.2012.10.016

90. Levine ME, Lu AT, Quach A, et al. An epigenetic biomarker of aging for lifespan and healthspan. *Aging*. 2018;10(4):573-591. doi:10.18632/aging.101414

91. Lu AT, Quach A, Wilson JG, et al. DNA methylation GrimAge strongly predicts lifespan and healthspan. *Aging*. 2019;11(2):303-327. doi:10.18632/aging.101684

92. Fann JR, Ribe AR, Pedersen HS, et al. Long-term risk of dementia among people with traumatic brain injury in Denmark: a population-based observational cohort study. *Lancet Psychiatry*. 2018;5(5):424-431. doi:10.1016/S2215-0366(18)30065-8

93. Nordström A, Nordström P. Traumatic brain injury and the risk of dementia diagnosis: A nationwide cohort study. *PLOS Med*. 2018;15(1):e1002496. doi:10.1371/journal.pmed.1002496

94. Loef M, Mendoza LF, Walach H. Lead (Pb) and the Risk of Alzheimer’s disease or cognitive decline: A systematic review. *Toxin Rev*. 2011;30(4):103-114. doi:10.3109/15569543.2011.624664

95. Schwartz BS, Stewart WF, Bolla KI, et al. Past adult lead exposure is associated with longitudinal decline in cognitive function. *Neurology*. 2000;55(8):1144-1150. doi:10.1212/WNL.55.8.1144

96. Reuben A. Childhood lead exposure and adult neurodegenerative disease. *J Alzheimers Dis*. 2018;64(1):17-42. doi:10.3233/JAD-180267

97. Genuis SJ, Kelln KL. Toxicant exposure and bioaccumulation: A common and potentially reversible cause of cognitive dysfunction and dementia. *Behav Neurol*. 2015;2015:e620143. doi:10.1155/2015/620143

98. Aloizou AM, Siokas V, Vogiatzi C, et al. Pesticides, cognitive functions and dementia: A review. *Toxicol Lett*. 2020;326:31-51. doi:10.1016/j.toxlet.2020.03.005

99. Silva PA, Hughes P, Williams S, Faed JM. Blood lead, intelligence, reading attainment, and behaviour in eleven year old children in Dunedin, New Zealand. *J Child Psychol Psychiatry*. 1988;29(1):43-52.

100. Reuben A, Caspi A, Belsky DW, et al. Association of childhood blood lead levels with cognitive function and socioeconomic status at age 38 years and with IQ change and socioeconomic mobility between childhood and adulthood. *JAMA*. 2017;317(12):1244-1251. doi:10.1001/jama.2017.1712

101. John P, Montgomery P. Does self-rated health predict dementia? *J Geriatr Psychiatry Neurol*. 2013;26(1):41-50. doi:10.1177/0891988713476369

102. Montlahuc C, Soumaré A, Dufouil C, et al. Self-rated health and risk of incident dementia: A community-based elderly cohort, the 3C Study. *Neurology*. 2011;77(15):1457-1464. doi:10.1212/WNL.0b013e31823303e1

103. Fischl B. FreeSurfer. *NeuroImage*. 2012;62(2):774-781. doi:10.1016/j.neuroimage.2012.01.021

104. Elliott ML, Caspi A, Houts RM, et al. Disparities in the pace of biological aging among midlife adults of the same chronological age have implications for future frailty risk and policy. *Nat Aging*. 2021;1(3):295-308. doi:10.1038/s43587-021-00044-4

105. d’Arbeloff T, Elliott ML, Knodt AR, et al. White matter hyperintensities are common in midlife and already associated with cognitive decline. *Brain Commun*. 2019;1(1). doi:10.1093/braincomms/fcz041

106. Elliott ML, Belsky DW, Knodt AR, et al. Brain-age in midlife is associated with accelerated biological aging and cognitive decline in a longitudinal birth cohort. *Mol Psychiatry*. Published online December 10, 2019:1-10. doi:10.1038/s41380-019-0626-7

107. Caspi A, Houts RM, Ambler A, et al. Longitudinal assessment of mental health disorders and comorbidities across 4 decades among participants in the Dunedin Birth Cohort Study. *JAMA Netw Open*. 2020;3(4):e203221-e203221. doi:10.1001/jamanetworkopen.2020.3221
